# Major roles of the circadian clock in cancer

**DOI:** 10.20892/j.issn.2095-3941.2022.0474

**Published:** 2023-01-12

**Authors:** Chen Huang, Chenliang Zhang, Yubin Cao, Jian Li, Feng Bi

**Affiliations:** 1 Department of Abdominal Oncology, Cancer Center and Laboratory of Molecular Targeted Therapy in Oncology, West China Hospital, Sichuan University, Chengdu 610000, China; 2 Laboratory of Molecular Targeted Therapy in Oncology, West China Hospital, Sichuan University, Chengdu 610000, China; 3 Department of Gastroenterology, West China Hospital, Sichuan University, Chengdu 610000, China; 4 West China School of Medicine, Sichuan University, Chengdu 610000, China

**Keywords:** Circadian clock, BMAL1, cancer, tumor therapy, oncology

## Abstract

Circadian rhythms are natural rhythms that widely exist in all creatures, and regulate the processes and physiological functions of various biochemical reactions. The circadian clock is critical for cancer occurrence and progression. Its function is regulated by metabolic activities, and the expression and transcription of various genes. This review summarizes the composition of the circadian clock; the biological basis for its function; its relationship with, and mechanisms in, cancer; its various functions in different cancers; the effects of anti-tumor treatment; and potential therapeutic targets. Research in this area is expected to advance understanding of circadian locomotor output cycles kaput (CLOCK) and brain and muscle ARNT-like protein 1 (BMAL1) in tumor diseases, and contribute to the development of new anti-tumor treatment strategies.

## Introduction

The circadian clock regulates various physiological processes and biochemical reactions in the human body. This system exhibits diurnal variation characteristics with time, which are denoted circadian rhythms^1^. Circadian rhythms are relatively conserved in evolution. They are widespread in various organisms and have evolved 4 times with selective advantages^2^. Because of its rhythm, the system is also referred to as the circadian clock system. This system comprises the central and peripheral clocks located in the hypothalamus’s anterior suprachiasmatic nucleus (SCN). The central and peripheral clocks have a synchronous circadian rhythm^3,4^. The central clock’s function can be performed independently, whereas the peripheral clock is coordinated through various signaling molecules to achieve synchronization.

Transcription-translation feedback loop (TTFL) is the molecular basis of circadian rhythms in organisms. TTFL regulates the central molecular circadian clock mechanism^1,5–7^. TTFL often plays a role in the SCN and peripheral tissues of the anterior hypothalamus of mammals. Optical signals are the main factors affecting the circadian clock. The optical signal stimulation received by optic nerve fibers generates downstream nerve or endocrine signal stimulation through the SCN, thereby synchronizing with peripheral organs^6–11^.

**Figure 1:**
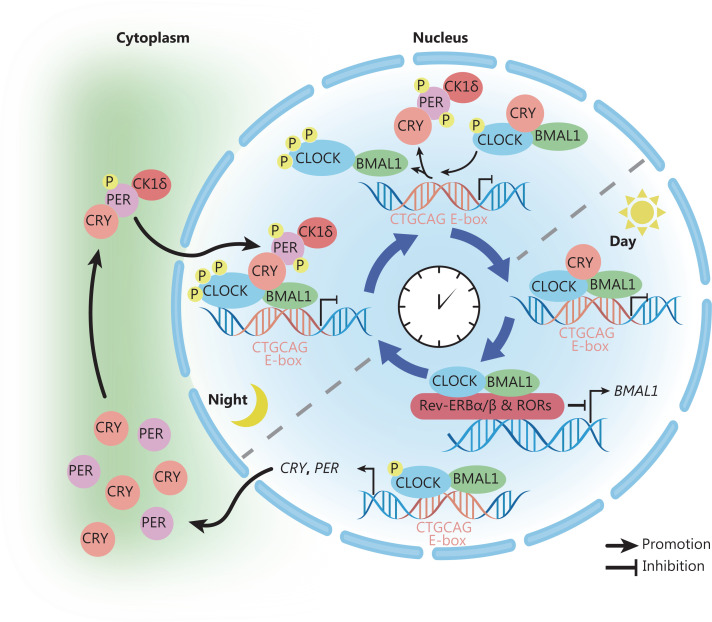
Molecular mechanisms of the circadian clock. The positive stimulus factor TTFL is composed of CLOCK, BMAL1, and its paralog NPAS2. This complex binds the target E-box (CTGCAG) sequence, thereby promoting the expression of transcription inhibitor CRY1/2 and PER1/2/3. CRY and PER serve as negative stimuli for TTFL. Two different stimuli form 2 complexes with opposite functions: the CLOCK-BMAL1 transcription activator and CRY-PER transcription inhibitor. Their growth and decline show a clear circadian rhythm. The CRY-PER complex enters the nucleus and inhibits the function of CLOCK-BMAL1 complex. the CLOCK-BMAL1 complex acts on the nuclear receptor Rev-ERBα/β (NR1D1/2) and RORs, and regulates their expression levels, thereby affecting BMAL1 expression and constituting the second feedback loop. Additionally, this loop is controlled by the kinase CK1δ/ε and ubiquitin ligases. TTFL, transcription-translation feedback loop; CLOCK, circadian locomotor output cycles kaput; BMAL1, brain and muscle ARNT-like protein 1; CRY, cryptochrome; PER, period; RORs, retinoic acid receptor-related orphan receptors. (In this picture, the dashed line indicates the boundary between the day and night phases, and the meaning of the remaining arrows is shown in the figure.)

The positive stimulus factor TTFL comprises circadian locomotor output cycles kaput (CLOCK), aryl hydrocarbon receptor nuclear translocator-like protein 1 (ARNTL), and its paralog NPAS2. ARNTL is also called brain and muscle ARNT-like protein 1 (BMAL1). TTFL binds the target sequence E-box (CTGCAG), thereby promoting the expression of transcription inhibitor cryptochrome (CRY1/2) and period (PER1/2/3), whereas CRY and PER serve as negative stimuli for TTFL^12–14^. Two different stimuli can form 2 complexes with opposite functions: the CLOCK-BMAL1 transcription activator and CRY-PER transcription inhibitor. Their growth and decline exhibits a clear circadian rhythm. As illustrated in **Figure 1**, the CRY-PER complex enters the nucleus and inhibits the function of the CLOCK-BMAL1 complex.

The above feedback loop is also consolidated by a secondary feedback loop. In the secondary feedback loop, the CLOCK-BMAL1 complex acts on the nuclear receptor Rev-ERBα/β(NR1D1/2) and retinoic acid receptor-related orphan receptors (RORs), and regulates their expression levels, thereby affecting BMAL1 expression^1,5^. Additionally, this loop is controlled by the kinase CK1δ/ε and ubiquitin ligases^15–20^. However, this basic understanding of the molecular mechanism underlying circadian rhythm in mammals is insufficient for routine medical care. Additional information is required regarding the mechanisms.

At the beginning of the circadian clock, owing to the presence of the negative regulatory factor CRY^12–14^, the CLOCK-BMAL1 complex cannot activate transcription even if it binds the E-box sequence. However, after a light period (midday of the circadian clock), CRY separates from the CLOCK-BMAL1 complex. Subsequently, because of the loss of CRY inhibition, transcription at the E-box is initiated, thereby promoting the expression of CRY and PER. Although PER continually accumulates, it cannot inhibit E-box transcription in the absence of CRY mediation. In addition, the CLOCK-BMAL1 complex acts on the nuclear receptor Rev-ERBα/β (NR1D1/2) and RORs and regulates their expression levels, thereby affecting the expression of BMAL-1^1,5^. At night, CRY and PER enter the nucleus in the form of CRY-PER-CK1δ, and PER mediates the phosphorylation of CK1δ on CLOCK. The new CLOCK-BMAL1 complex with CRY replaces the complex that bound the E-box in the previous phase. This inhibition differs from that mediated by CRY. Finally, the CLOCK-BMAL1 complex combined with CRY binds the E-box before a new cycle begins^21^.

Several studies have revealed a close relationship between circadian rhythm disorders and cancer. A meta-analysis of various human cancer transcriptome datasets has revealed the dysregulated expression of circadian genes in different tumor types^22^. First, a mutual regulatory mechanism exists between circadian and cancer genes. The genes regulated by the circadian clock include various oncogenes and tumor suppressor genes, and the core genes of the circadian clock are also regulated by oncogenes and tumor suppressor genes, which are involved in tumor onset and malignancy^23,24^. Second, the circadian clock regulates gene rhythms associated with metabolic and endocrine functions, and metabolic activity and endocrine homeostasis play important roles in tumor development^25–28^. In addition, abnormal biological rhythms promote the malignant progression of tumors by weakening the body’s immunity. Immunity is an important factor restricting tumor development. Disrupting biological rhythms not only affects the innate and acquired immunity of the body, but also promotes tumor immune escape through immune checkpoints^29–32^. In summary, the circadian clock may play an important role in the entire process of tumorigenesis, and has the potential to be applied in tumor prevention, diagnosis, and treatment.

## Methods

In the analysis accompanying this review, RNA-seq data (level 3) and corresponding clinical information on pan-cancer were obtained from the Cancer Genome Atlas (TCGA) database (https://portal.gdc.com). For the predictive analysis of single genes in multiple tumors, univariate Cox regression analysis and a forest plot constructed with the “forest plot” R package revealed the *P*-values, HR, and 95% CI. Tumor mutation burden (TMB) was derived from The Immune Landscape of Cancer, published by Vesteinn Thorsson in 2018^33^. Immune-associated assessment was performed with xCell analysis. All bioinformatics analyses were performed in R software v4.0.3 for statistical analysis. If no additional explanation is provided, the rank-sum test was performed to detect the differences between the 2 groups of data, and *P* < 0.05 was considered statistically significant.

## Circadian clock and cancer

Human genes are highly similar to mouse genes. Previous studies have found that nearly half the protein-coding gene expression in mice is associated with the circadian rhythm. The circadian clock controls the rhythmic expression of some genes in organisms under TTFL regulation, and these genes further regulate the expression of other genes. A total of 50%–80% of protein-coding genes in mice and humans have been found to show rhythmic oscillations^5^. The circadian clock is widely expressed in human genes and is influenced by environmental factors (light and food). When the circadian clock rhythm is perturbed by various factors, the normal biological characteristics of human organs or cells are altered. These changes may be correlated with the pathogenesis of various diseases, including some psychological diseases, endocrine diseases, and even cancer^7,10,34–39^. The physiological activities, cell metabolism, proliferation, and differentiation in the human body all show rhythmicity. Cancer cells, which abnormally proliferate in the human body, may be more susceptible to circadian clock disorders than normal cells because of their unstable characteristics.

**Figure 2:**
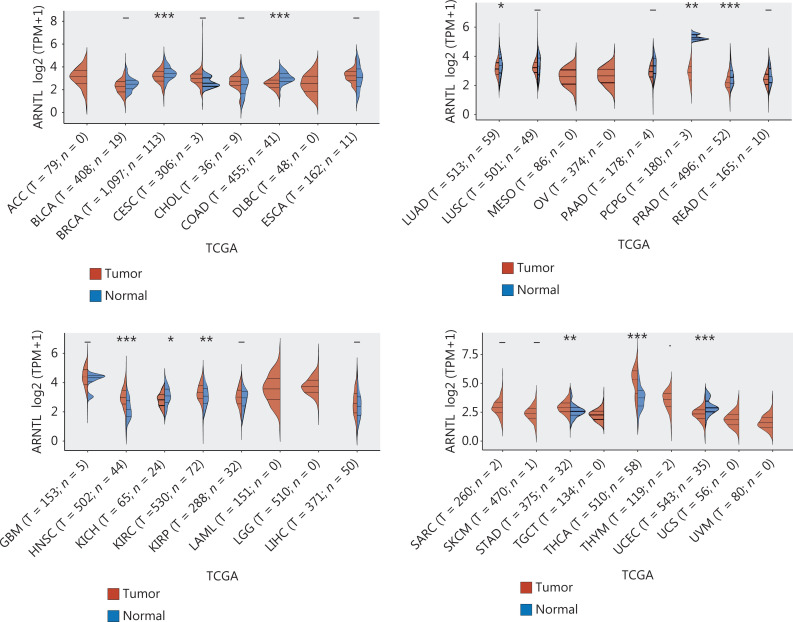

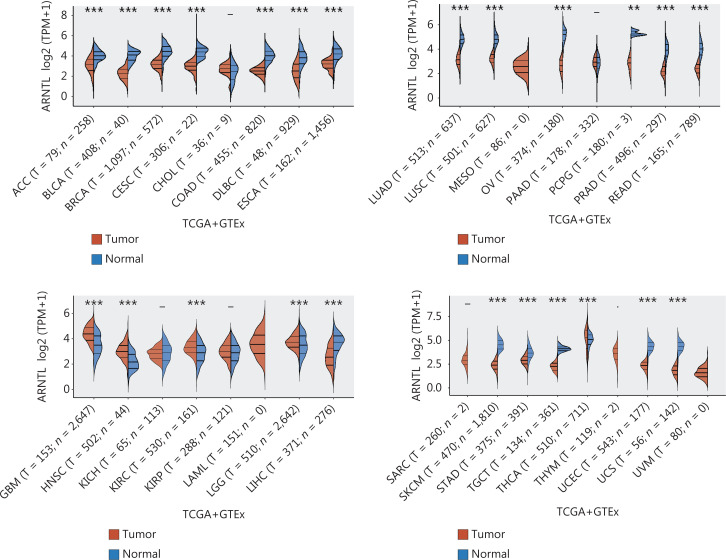
Expression analysis of BMAL1 in tumor tissues. The expression distribution of the BMAL1 (ARNTL) gene in tumor and normal tissues. The abscissa represents different tumor tissues, and the ordinate represents the gene expression distribution. Different colors represent different groups. *P < 0.05, **P < 0.01, ***P < 0.001, with asterisks indicating significance. The Wilcoxon test indicated that the 2 sample groups were dominant.

Are circadian clock disorders associated with cancers? At present, the controversy regarding this issue has related primarily to several earlier epidemiological studies and recent studies on the clear correlation between clock disorders and cancers. Previous epidemiological investigations have revealed a lack of correlation between circadian clock disorders and the development of specific cancers. However, according to the International Agency for Research on Cancer (IARC) and other recent research, when the human circadian clock is disrupted, the likelihood of developing cancer, including lung cancer, intestinal cancer, and breast cancer, dramatically increases^5,40–46^. The above controversy may be due to the use of different research methods, or differences in sample size and representativeness. In clinical practice, the influence of circadian rhythms on human malignant tumors has been observed. Lou et al.^47^ have investigated thyroid nodules in older people with varying degrees of malignancy, and concluded that poor sleep quality and biological rhythm disorders are independent risk factors. CLOCK and BMAL1 expression levels were found to be considerably higher in the malignant thyroid nodule group, whereas CRY2 expression was significantly lower. In addition, with the popularity of next-generation sequencing, bioinformatics technology has provided further evidence. The bioinformatics analysis depicted in **Figure 2** supports a correlation between clock disorders and cancers. In different cancers, BMAL1 expression significantly differs between tumor and normal tissues. PER and CRY expression are detailed in the Supplementary Materials.

In addition to the above evidence, animal experiments have demonstrated a relationship between circadian rhythms and cancer. When CLOCK and BMAL1 mutations are artificially introduced, premature aging has been observed in mice that are not predisposed to cancer^48,49^. According to Papagiannakopoulos^50^, prolonged jet lag dramatically exacerbates cancer development and progression in K-Ras and p53 deficient mice.

## The mechanisms of the circadian clock in cancer progression

We investigated the mechanisms underlying the effects of the circadian clock in cancer and assessed their similarities. The mechanisms may include the following: (1) Tumor cells undergo a variety of biochemical reactions, including cell growth and senescence, cell proliferation and apoptosis, DNA damage repair process, and various metabolic processes^1,5,36,44,51–55^; the circadian clock may affect tumor occurrence and development by regulating these reactions^44,56^. (2) Cancer stem cells (CSCs) undergo tumorigenesis, development, and metastasis, and the circadian clock plays a crucial role in the stemness of self-renewal cancer cell subsets, such as acute myeloid leukemia (AML) and pleomorphic glioblastoma (GBM)^57–60^. (3) CLOCK components regulate the expression of angiogenic factors such as hypoxia-inducible factor 1α (HIF-1α), aryl hydrocarbon receptor nuclear translocator (ARNT), and vascular endothelial growth factor (VEGF) in cancer cells. Increased levels of these angiogenesis-promoting factors in the tumor microenvironment (TME) promote tumor development and metastasis^43,61–63^. (4) Previous research indicates that CLOCK regulates the inflammation mediated by myeloid cells—a crucial cancer marker^63,64^. For instance, in GBM, CLOCK changes the microglial content of GSC through transcriptional regulation of the chemokine olfactomedin-like 3^58^. The infiltration of immune cells such as macrophages and neutrophils in renal clear cell carcinoma is associated with rhythmic changes in the expression of CLOCK-associated components (BMAL1, PER, etc.)^52,65^.

**Figure 3:**
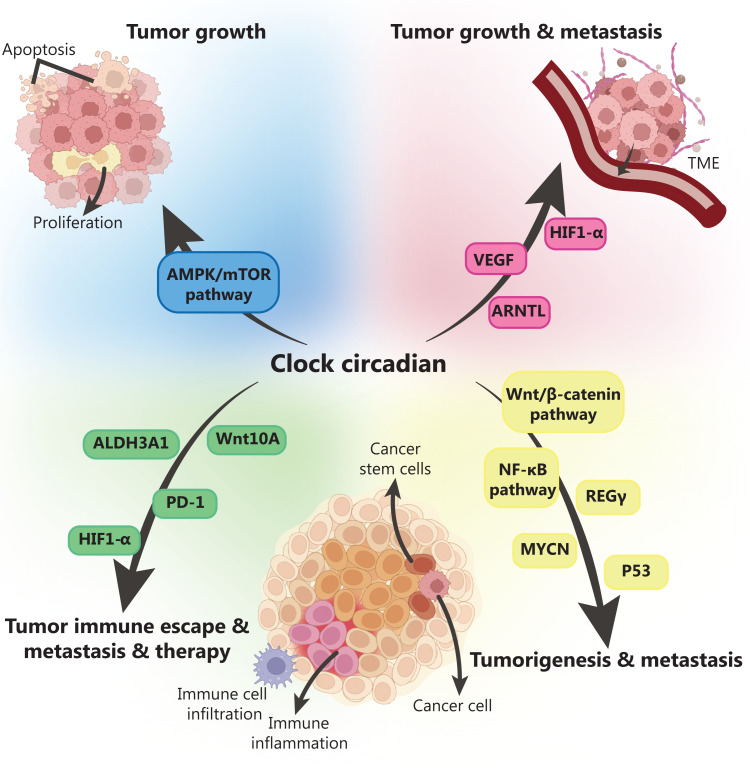
Mechanisms of the circadian clock in cancer. The circadian clock plays an important role in tumorigenesis, tumor growth, metastasis, tumor immune escape, and other processes by regulating various biological functions, such as apoptosis and proliferation. Among them, many signaling pathways, such as the AMPK/mTOR pathway, Wnt/β-Catenin pathway, and NF-κ B pathway, as well as molecules, such as Hif-1α, P53, and PD-1 may play important roles.

In addition, immune escape is an integral part of cancer progression^63^. According to Chen et al.^58^, changes in CD8^+^ T cells often affect CLOCK expression in patients with glioblastoma multiforme. In the 4T1 mouse model of breast cancer, the CLOCK component has been found to induce regular expression of Wnt family member 10A (Wnt10A) and upregulate the expression of downstream acetaldehyde dehydrogenase 3 (ALDH3A1). ALDH3A1 is a characteristic of CSCs and is associated with the degree of tumor malignancy^66,67^. Moreover, previous studies have indicated that the depletion of T cells and up-regulation of programmed death-ligand (PD-1) in patients with cancer may be associated with the widespread mutation and genomic instability of the CLOCK gene^32,52^. This evidence suggests that CLOCK gene expression in cancer cells may contribute to immune escape. **Figure 3** shows these relationships more intuitively.

## CLOCK and the potential molecular mechanisms of tumorigenesis

### Ghrelin

Ghrelin is a 28-amino acid peptide that acts a ligand for GHSR. It activates and promotes appetite, and affects many physiological activities, such as glucose metabolism and gastric acid secretion^68–70^. Previous studies have indicated the role of Ghrelin in tumor cells, such as those in rectal cancer, liver cancer, leukemia, endometrial cancer, and pancreatic cancer^71–75^. Regulation of the GHS-R/Ras/PI3K/Akt/mTOR pathway may be one of Ghrelin’s mechanisms, particularly in colon cancer^76^. GLUT1 may be another potential target. Previous studies have confirmed that Ghrelin promotes oral cancer progression through GLUT^77^. However, Ghrelin notably has a dual role in prostate cancer, and whether it promotes tumor occurrence and development remains unknown^78,79^.

According to Segers et al.^80^, feeding time and BMAL1 gene expression have circadian rhythms, thus leading to periodic changes in short-chain fatty acids, and consequently affecting the release of Ghrelin. Ghrelin decreases to its lowest level within 1 hour after a meal and exhibits diurnal variation with the circadian rhythm^81–83^. After knockout of the BMAL1 gene in mice, the circadian rhythm fluctuation of Ghrelin disappears, thus suggesting a specific relationship between BMAL1 and Ghrelin; these rhythm-associated characteristics may be associated with tumorigenesis^84^.

### Unfolded protein response (UPR)

The UPR is a stress response caused by protein accumulation in the endoplasmic reticulum, owing to abnormal protein folding into secondary structures. Additionally, it is a cell checkpoint pathway triggered by incorrect folding of the endoplasmic reticulum. The UPR involves Protein kinase RNA-like ER kinase (PERK), Activating Transcription Factor 6 (ATF6), and Inositol-requiring enzyme 1 (Ire1)^85^.

miR-211 is a protein kinase RNA-like ER kinase-induced micro-RNA. According to Bu et al.^86^, miR-211 regulates BMAL1 expression and CLOCK composition. BMAL1 inhibition impairs UPR-dependent protein synthesis and affects the adaptation of cells to cell stress caused by endoplasmic reticulum homeostasis damage. Moreover, Bu et al. have discovered that the UPR inhibits BMAL1 in Burkitt lymphoma, thereby affecting circadian rhythm and protein synthesis, and promoting tumor progression, which may be associated with the continual activation of c-myc. Knockout of PERK in c-myc-driven lymphoma has been found to lead to ER protein overload and cancer cell death^87^. Inhibition of BMAL1 by PERK-miR-211 is crucial for promoting the progression of c-myc-positive lymphoma. These results demonstrate that PERK-induced miR-211 inhibits BMAL1 expression, limits protein overload, and promotes tumor progression.

### Effects on anti-tumor immunity by regulation of TAM metabolism via Hif-1α

Hif-1α is a major factor regulating glycolysis and the synthesis of metabolic gene transcription and is also crucial for the synthesis of arginase-1 (Arg-1), a urea-cycling enzyme^88–90^. Previous research has indicated that tumor-associated macrophages (TAMs) upregulate Arg-1 through Hif1-α^84^, a macrophage marker. Anti-tumor immunity mediated by cytotoxic T-cell nuclear natural killer cells is an important factor affecting tumor progression. Arginine depletion in TAMs inhibits this process, which is associated with the abnormal expression of Arg-1^91,92^.

We previously discovered that fluctuations in circadian rhythm regulate the inflammatory function of macrophages. Knockout of BMAL1 increases mortality in septic mice. BMAL1 controls the rhythm of monocyte recruitment, thus playing a crucial role in the immune response to pathogens^93^. Alexander et al.^94^ have demonstrated that macrophages induce BMAL1 expression after stimulation (inflammation, etc.), thereby aggravating Hif-1α-dependent metabolic reprogramming and causing mitochondrial dysfunction. The balance between BMAL1 and Hif-1α affects the metabolism of macrophages and anti-tumor immunity. BMAL1 gene deletion promotes tumor growth.

### Myc

Oncogenes are genes that cause malignant transformation of cells. Dysfunction in the major oncogene family Myc or abnormal proliferation often leads to the occurrence and adverse prognosis of different tumors^95–97^. Myc and its partner MAX form E-box motifs similar to those of the BMAL1 gene that control circadian rhythms. Therefore, Myc also participates in cell growth, proliferation, and death^98,99^.

Previous studies have reported that Myc enhances the transcriptional activity of E-box sites and increases REV-ERBα/β expression, thus inhibiting BMAL1 function^100^. Myc expression is negatively correlated with the circadian clock^23^. As described above, PER is a negative stimulus of TTFL, and the CRY-PER complex inhibits the function of the CLOCK-BMAL1 complex. This inhibitory effect of PER has been proposed to be achieved through Myc^101–103^.

According to Anastasia et al.^104^, c-Myc plays a major role in CLOCK oscillators. Myc expression has a strict circadian rhythm, but its expression also responds to CLOCK function. Concurrently, PER and BMAL1 inhibit the transcription of c-Myc during tumorigenesis and development. Deletion of either of these 2 genes increases c-Myc expression, as demonstrated in a mouse lung cancer model^50^.

### Ras Homolog Family Member A (RhoA)

The Rho family is involved in the occurrence and development of various tumors. Its GTPase activity regulates cell transformation, division, and angiogenesis^105^. RhoA regulates the adhesion, aggregation, and morphology of various cells by controlling actin and myosin contraction^106,107^. ROCK is the downstream effector of RhoA, and cofilin (CFL) is an actin-depolymerizing factor. Ma et al.^108^ have demonstrated that CLOCK and BMAL1 regulate the dynamic conversion of F-/G-actin by controlling the RhoA-ROCK-CFL pathway, thereby promoting the proliferation and invasion of tumor cells, which may be associated with inhibition of CUL3-mediated ubiquitination and an increase in RhoA expression.

### Protein kinase

Several kinases regulate Clock/Bmal and other circadian genes, such as AMP-activated protein kinase (AMPK), Akt, and mammalian rapamycin target protein (mTOR). Their roles are crucial for cancer development. Protein kinases involved in post-translational modifications play important roles in regulating the biological clock, and protein kinases may be involved in the occurrence and development of various diseases caused by biological clock disorders, including tumors. Ramanathan et al.^109^ have found that inhibition of mTOR function slows circadian rhythms, thus suggesting biological regulation of mTOR in circadian rhythms. mTOR, a central regulator of multiple tumor-associated signaling pathways, plays important roles in cell proliferation, growth, differentiation, and survival. Abnormal activity of mTOR is closely associated with the occurrence, development, metastasis, and drug resistance of various malignant tumors, including lung cancer^110,111^. The PI3K/Akt/mTOR signaling pathway regulates cell growth and proliferation. Abnormal activation of this pathway is closely associated with tumor occurrence and development^112^. The AMPK/mTOR signaling pathway is involved in tumor cell proliferation, apoptosis, invasion, metastasis, and drug resistance^110^. AMPK is an important cellular energy sensor that plays a role in metabolic control and serves as a central sensor for metabolic signals^113^. On the one hand, AMPK-mediated phosphorylation of CRYs and CK1 regulates the negative feedback control of the circadian clock through proteolytic degradation, thereby shortening the rhythm cycle^114,115^. On the other hand, most early studies have suggested that AMPK itself acts as a tumor suppressor in tumors. AMPK activation is conducive to the occurrence of various malignant tumors by inhibiting the proliferation of tumor cells, and it also promotes apoptosis by inhibiting the mTOR signaling pathway^116,117^. In summary, various protein kinases and biological clocks have close and complex relationships in tumor development.

### Other possible mechanisms

In addition to the potential relationship between CLOCK and tumors, various studies have suggested potential mechanisms that may have research value. For instance, in the TME in prostate cancer, if PER expression is too low, BMAL1 expression increases, thus resulting in up-regulation of β-Catenin phosphorylation and activation of the Wnt/β-Catenin pathway. PER is a negative regulator of prostate cancer stem cells and is used as a new target for prostate cancer treatment^118,119^. In other studies, some potential targets have been proposed but have not been studied, such as iron death (colon cancer), NF-κB, REGγ, MYCN (advanced neuroblastoma), and P53^120–124^. Length constraints prevent us from stating them individually herein. These mechanisms may require additional research to guide the development and exploration of related cancer treatment issues.

## Effects of CLOCK components in specific cancers

**Figure 4:**
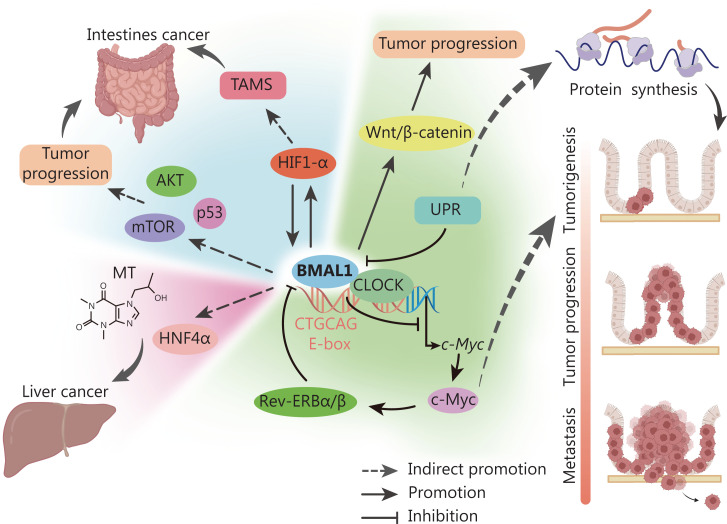
Functions and effects of BMAL1 in cancer. The known and potential mechanisms of BMAL1 are involved in all cancer stages, including protein synthesis, tumorigenesis, tumor progression, and metastasis. This figure was created with biorender.com.

In addition to the aforementioned mechanisms, CLOCK may exhibit different biological functions and characteristics in different cancers (**Figure 4**). Here, we briefly discuss the roles of CLOCK/BMAL1 in in several representative cancers.

### Hepatocellular carcinoma (HCC)

HCC is one of the most common malignant tumors and the fourth leading cause of cancer-associated deaths worldwide^125^. Because of its low detection, high recurrence rates, and poor prognosis in the curable stage, the survival rate of patients is unsatisfactory despite recent progress in treatment strategies^125^. In 2012, approximately 700,000 people died from liver cancer. According to the World Health Organization, more than 1 million people will die of liver cancer in the year 2030^126,127^. The main risk factors for liver cancer include hepatitis C or hepatitis B viral infection, long-term alcohol consumption, and nonalcoholic fatty liver disease (NAFLD), including the effects of smoking, obesity, diabetes, and excessive iron load^128^.

HCC, the most common type of liver tumor, is found in approximately 90% of patients with a cirrhosis history^126,129^. Previous studies have demonstrated that time-caloric restriction upregulates the expression of BMAL1, limits the transformation of liver fibrosis to cirrhosis, and further leads to HCC development, as verified in a liver cancer model induced by diethylnitrosamine^130^. These findings suggest that CLOCK may influence liver cancer occurrence by affecting liver cirrhosis progression.

Metallothionein (MT) expression controlled by CLOCK is associated with various endocrine diseases, mental diseases, and tumor diseases in humans. MT expression is relatively extensive in the liver and is regulated by metal regulatory transcription factor-1 (MTF-1)^131–133^. MT expression is a prognostic indicator in patients with HCC^121^. Interactive relationships among CLOCK, MT, and HCC have been identified. Li et al.^134^ have confirmed that disorders in MTs and circadian clock genes play crucial roles in human HCC, possibly because of their influence on the expression of nuclear orphan receptor factor protein (Nr1d1) and D-box binding protein (DBP). Concurrently, melatonin, a drug used to treat various diseases and inhibit tumor growth^135–137^, prevents BMAL1 expression, an effect potentially associated with the upregulation of MT receptors and the decrease in Hif-1α^138^.

In addition, Hepatocyte nuclear factor 4α (HNF4α) is a cytokine widely present in the nuclei of hepatocytes, which is involved in the metabolic processes of various hepatocytes^139,140^. HNF4α has various physiological functions, including inhibiting expression of multiple genes (such as cyclin D1 and CCND1) in HCC development^141–146^. HNF4α expression may be negatively correlated with tumor growth, and its overexpression may inhibit liver cancer growth. Similarly, HNF4α expression is relatively lacking in human HCC^145,147^. HNF4α has 2 different promoters, P1 and P2, producing HNF4α1/2 and HNF4α7/8, respectively. Notably, this difference also exists in colon cancer^142,148,149^. According to research by Fekry and colleagues^150^, both subtypes have an apparent circadian rhythm, but P2-HNF4α directly inhibits BMAL1 expression, thereby increasing P1 isoform expression in the cytoplasm. In HNF4α-positive HCC, the increase in BMAL1 expression slows tumor growth *in vivo*, thus indicating that BMAL1 may be a potential target for reversing HNF4α-positive HCC.

### Colorectal cancer (CRC)

CRC is a common malignant tumor. CRC-associated deaths account for approximately 10% of cancer-associated deaths worldwide and are the major cause of cancer deaths^151–153^. Circadian rhythm is an important factor affecting CRC occurrence and development. CLOCK inhibits early tumorigenesis in the intestines. BMAL1 affects AKT, mTOR, and P53^154^, and regulates various signal transduction pathways in intestinal stem cells (including the Hippo pathway), thus affecting CRC occurrence^155^. As described above, BMAL1 influences tumor progression by regulating the inflammatory response, in agreement with the findings of Liu et al.^156^ indicating that circadian rhythm disruption leads to impaired regulatory B cell function and apoptosis of CD4^+^ T cells in the intestinal epithelium, and subsequently enteritis progression and CRC occurrence.

Liver metastasis of CRC (CRLM) is a sign of poor prognosis in CRC, and lung and liver metastases are among the leading causes of CRC-associated deaths^157^. CLOCK expression is perturbed in CRLM model mice^158^, thus suggesting the effects of CLOCK-associated components on CRLM. BMAL1 also activates Rab27a, a key molecule associated with exosome secretion, and affects CRLM by enhancing exosome secretion in CRC cells^159^.

### Breast cancer

Breast cancer is the second leading cause of cancer-associated deaths globally and the most common cancer in women^160,161^. In breast cancer cells, compared with normal breast cells, previous studies have found that the circadian rhythm and CLOCK gene expression show different degrees of damage^162,163^, and the expression of core CLOCK genes in patients with breast cancer is altered, thus confirming the role of CLOCK in breast cancer^164,165^. BMAL1 knockout promotes breast cancer cell metastasis^166^ and decreases acidosis-mediated metastasis of breast cancer by decreasing hypoxia-induced acidosis in the TME^167^, thus providing a potential mechanism for preventing breast cancer metastasis.

Triple-negative breast cancer (TNBC) is a type of breast cancer with high invasiveness, poor differentiation, and ready metastasis, which accounts for about 15%–20% of all breast cancer. Owing to a lack of ER, PR, and HER-2, treatment is limited, and TNBC prognosis is poor^168^. However, studies have confirmed that acetylserotonin methyltransferase (ASMT) affects the invasiveness of TNBC cells by regulating circadian rhythms. Thus, ASMT is currently the most promising target for TNBC treatment^169^. According to recent research, BMAL1 may be directly or indirectly affected by insulin and feeding, thus resulting in changes in the characteristics of TNBC, an aspect warranting further attention^65,170^.

### Glioma

Glioma is a primary tumor of the central nervous system. As described earlier, in addition to the important roles of CSCs and CD8+ T cells in glioma, CLOCK alters the content of microglia in GSC through transcriptional regulation of the chemokine olfactomedin-like 3. In the tumor microenvironment of glioma, glioma-associated microglia (GAMs) are key components regulating tumor progression^171^. GAMs are divided into 2 subtypes with opposite functions: M1 and M2, which inhibit and promote tumor progression, respectively^172–174^. Exosome miRNA is an important medium for communication of various biological information inside and outside cells. Li et al.^175^ have used exosome-associated technologies to confirm that M2 subtype GAMs regulate the expression of tumor-associated proteins and decrease apoptosis, thereby promoting the proliferation and metastasis of glioma, which is associated with the recruitment of miR-7239-3p and a decrease in BMAL1 expression in TAMs.

The circadian clock has a wide range of effects on specific cancers. Because of space limitations, we discuss several representative cancers. Other factors such as AML^60^ and melanoma^176,177^ have been associated with CLOCK. However, the specific mechanisms remain unclear or controversial, and require further investigation.

## Circadian clock and cancer treatment

We have described the complicated mechanisms underlying the relationship between circadian rhythm and cancer. To some extent, these mechanisms are believed to be valuable in guiding cancer treatment. Both *in vivo* and *in vitro* experiments and statistical studies on clinical cases have shown that biological CLOCK has important research value in treating tumors or regulating the anti-tumor efficiency of radiotherapy and chemotherapy^178–181^. Several aspects are summarized below.

### CLOCK affects the sensitivity of tumor cells to treatment and the efficacy of drugs

As described previously, CLOCK affects the occurrence, development, and metastasis of CRC. The standard chemotherapy agents 5-fluorouracil and bevacizumab (Beva) are often used for metastatic CRC. Bevacizumab inhibits angiogenesis by preventing the binding of vascular endothelial growth factor A (VEGFA) to its receptor, thereby achieving antitumor therapeutic effects. However, it is effective for only some clinical patients, and its anti-angiogenic effect is adaptive^182,183^, thus suggesting the possibility of drug resistance. BMAL1 upregulates the promoter activity of VEGFA and enhances its expression, as confirmed by BMAL1 inhibition^184–186^. In addition, VEGF expression in xenografts (sarcomas, melanomas, etc.) is induced by hypoxia. Circadian rhythms are inhibited by transcription of PER2 and CRY1. Therefore, ZT2 is more effective in anti-angiogenesis therapy than ZT14 (where ZT0 is bright, and ZT12 is dark)^184^. After administration of Beva and other anti-angiogenic drugs, BMAL1 expression in cancer cells increases, thus leading to the activation of VEGF expression and inducing drug resistance^187^. These results emphasize the roles of CLOCK in influencing tumor angiogenesis and anti-angiogenic drug resistance.

Epithelial-mesenchymal transition (EMT) refers to the polarization of epithelial cells to obtain characteristics of mesenchymal cells. This process enhances the migration and invasion of cells, and mediates drug resistance. EMT is the first key step in metastatic CRC development^188^. In the formation and maintenance of EMT, the downregulation of E-cadherin expression causes functional disorders of the E-cadherin/β-catenin complex, and various signaling pathways (such as TGF-β and Wnt) are involved^189,190^. A recent report^191^ has indicated that BMAL1 is critical in EMT-induced CRC metastasis and drug resistance to chemotherapeutic drugs. Understanding the importance of BMAL1 in the epithelial-mesenchymal equilibrium of CRC cells will aid in studying how to reverse drug resistance to existing treatment methods. Similar findings have been reported for breast cancer and glioma.

A study on MCF10A (non-tumorigenic) and MDA-MB-231 (invasive-tumorigenic) cells with 2 different tumorigenic characteristics of human breast epithelial cells has found that knockout of BMAL1 increases sensitivity to cisplatin and doxorubicin^23^. Temozolomide (TMZ) is a DNA alkylator commonly used to treat GBM. When the effect of TMZ on GBM (including DNA damage, cell death and tumor growth inhibition) reaches the maximum daily peak of BMAL1 expression, the anti-tumor effect of TMZ has circadian regularity, which may be associated with differing sensitivity of GBM cells to TMZ, owing to their circadian regularity^192^. Similarly, in a study of GBM cells, after bortezomib treatment, cell viability has been found to change with time, and this rhythm is altered by BMAL1 knockdown^193^.

In summary, the destruction of BMAL1 usually increases the sensitivity of tumor cells to chemotherapeutic drugs; however, some studies have indicated opposite results. For example, in pancreatic cancer^194^, BMAL1 is targeted and inhibited by miR-135b, thus resulting in local circadian rhythm disorders. However, this destruction has been found to cause tumor resistance to chemotherapeutic drugs, in contrast to previous conclusions. Similarly, in tongue squamous cell carcinoma, BMAL1 overexpression increases sensitivity to paclitaxel, whereas decreasing the expression of BMAL1 induces drug resistance in tongue squamous cell carcinoma cells^195^.

Some researchers have proposed that 1A-116 in GBM treatment, at the end of light (ZT12) administration rather than the beginning of light (ZT3) administration, prolongs survival time in mice^196^. This finding is contrary to the conclusion that at ZT2, the time of administration in CRC, anti-angiogenesis drugs have a more potent anti-tumor effect. Treatment with 1A-116 specifically inhibits the activation of Ras-related C3 botulinum toxin substrate 1 (RAC1) in GBM cells, thereby affecting the role of RAC1 in tumor progression. The mechanism of action of 1A-116 may block the binding of RAC1 to guanine exchange factor by interacting with Trp56 residues and interfering with the normal biological function of RAC1^197–200^. This contradictory phenomenon requires further research.

Beyond increasing the sensitivity of tumor cells to anti-tumor drugs, some drugs, such as doxorubicin, destroy circadian cell rhythms by inhibiting BMAL1 expression^201^. Thua, targeting BMAL1 may be a feasible strategy for improving the therapeutic effects of anti-tumor drugs. These CLOCK-mediated differences in the sensitivity of anti-cancer drugs may be used to optimize and adjust treatment regimens and medication protocols to achieve the best anti-tumor effect. However, BMAL1 has a bidirectional role: decreasing BMAL1 expression increases the sensitivity of cells to anticancer drugs, but also enhances the invasiveness of tumors and causes distant metastasis. Therefore, this conclusion should be comprehensively considered.

## Potential therapeutic targets or drugs

We described some potential mechanisms through which CLOCK affects cancer progression. The components associated with these mechanisms have the potential to be used as targets for cancer treatment. Some drugs may have anti-tumor effects by regulating circadian rhythms, as summarized below.

### Ghrelin

Cachexia occurs in most cancer patients, particularly in the terminal stage of cancer and in patients with metastasis. It is a severe cancer complication that directly affects patients’ tolerance to treatment, and can even cause disability or death^202–204^. More than half of all patients with cancer are affected by cachexia, and at least 20% of patients with advanced cancer die^205^. As described above, Ghrelin, an intestinal hormone, activates and promotes appetite and affects glucose metabolism, gastric acid secretion, and other physiological activities. Consequently, it is often considered to reverse cancer-associated cachexia and to be a promising treatment^206–209^. The rhythmic characteristics of Ghrelin secretion may provide a more optimized solution for future clinical applications.

### CLK8

According to Doruk et al.^210^ CLOCK’s interaction with small molecules (CLK8) decreases the interaction between CLOCK and BMAL1 and interferes with the translocation of CLOCK into the nucleus. This decrease in CLOCK in the nucleus leads to a more stable negative arm of TTFL and enhances the role of the circadian clock. Therefore, by influencing the functions of CLOCK and BMAL1, CLK8 elicits improvements in aging, emotional disorders, and the effectiveness of cancer treatment. Another small molecule, Nobiletin, a natural polymethoxy flavonoid compound, may also enhance the CLOCK effect by increasing the PER2 level and consequently stabilizing the negative arm of TTFL.

The above small molecule substances may enhance the functions of CLOCK and BMAL1, and are the leading compounds and potential targets for preventing and treating new therapies for diseases associated with circadian rhythm changes, such as cancer.

### mTOR

mTOR is a highly conserved serine/threonine-protein kinase associated with cell proliferation, protein metabolism, and autophagy^211–214^. The mTOR signaling pathway is abnormally expressed in various tumors. Although mTOR inhibitors have broad application prospects in antitumor therapy, problems remain, such as low treatment efficiency and easy recurrence.

Ramanathan et al.^109^ have found that inhibition of mTOR function slows circadian rhythms, thus suggesting biological regulation of mTOR in circadian rhythms. The PTEN gene inhibits tumor growth by negatively regulating PI3K and its downstream targets (mTOR/AKT, etc.). PTEN dysfunction is observed in melanoma, breast cancer, liver cancer, and other cancers. According to previous research^215^, targeting PTEN upregulates BMAL1 expression, whereas this effect is reversed by rapamycin (mTOR inhibitor). The accumulation of BMAL1 caused by PER deficiency is also eliminated by rapamycin. Thus, mTOR-Bmal may be a potential clinical target. Combined biological rhythm and anti-mTOR drugs may further improve the anti-tumor effects of traditional anti-mTOR drugs.

### UPR

Metabolic disorders, viral infection, hypoxia, and tumors in the body usually cause UPR, and irreparably damaged cells may undergo apoptosis, on the basis of decreased protein synthesis. The UPR is often involved in tumor occurrence and development. As described above, it promotes the survival of tumor cells, which may be associated with the inhibition of BMAL1 expression by miR-211 induced by PERK^87,216–218^. Therefore, PERK and Ire1, as UPR transducers, are potential targets for treating tumor progression.

### HSP90

The expression of some HSP90 isomers may have a circadian rhythm. Although the amplitude of this rhythm is low, it nonetheless affects the cell cycle process and may lead to time-dependent anti-tumor efficacy of some drugs. Therefore, targeting HSP90 also improves therapeutic effects^219^.

### MLN4924

Osteosarcoma is a malignant bone tumor that occurs primarily in adolescents. Surgery after neoadjuvant chemotherapy is a conventional treatment, and adjuvant chemotherapy (cisplatin, doxorubicin, etc.) is performed after surgery. The 5-year survival rate remains low^220,221^. Nedd8 is a ubiquitin-like molecule with a molecular weight of approximately 8 kDa whose substrate is cullin protein. Cullin protein, as the scaffold of Cullin-Ring E3-ubiquitin ligases, participates in the degradation of cellular proteins in the ubiquitin-proteasome system. MLN4924 (Pevonedistat) is an inhibitor of Nedd8 activation enzyme, which has anti-cancer effects and inhibits the occurrence and development of tumors^222–227^. According to Zhang et al.^228^, MLN4924 is an effective drug for treating osteosarcoma and has vast application prospects. RORα and BMAL1 may mediate its inhibitory effect on tumor growth.

## Focus on therapeutic approaches associated with circadian-mediated immune responses

Lymphocytes often play important roles in antitumor immunity, and their biological activity is often regulated by CLOCK. Many cytokines with immune activity have a clear circadian rhythm during their production or secretion. For example, CD4^+^ 17 helper T cells (Th17) produce interleukin-17 (IL-17), and Th17 differentiation is regulated by the transcription factor RORγT, which has a circadian rhythm^229^. RORγ activation enhances the cell differentiation and biological function of Th17 and decreases the Treg level (But Treg itself has no circadian rhythm^230^), thus inhibiting tumor growth. This antitumor effect has also been verified in animal models^231^.

We also suggest that the circadian clock affects anti-tumor immunity by regulating immune-associated functions, such as depletion of T cells, enhancement of immune escape, and controlling the metabolic process of TAMs by affecting the function of Hif-1α. Therefore, focusing on these immune responses associated with CLOCK and BMAL1 should provide new insights into cancer treatment.

**Figure 5:**
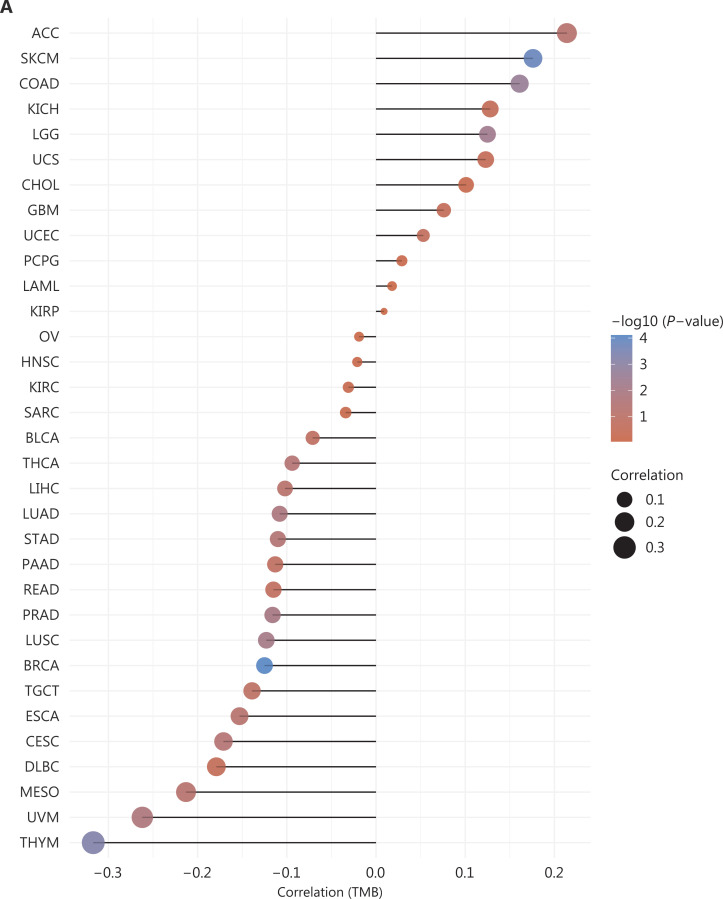

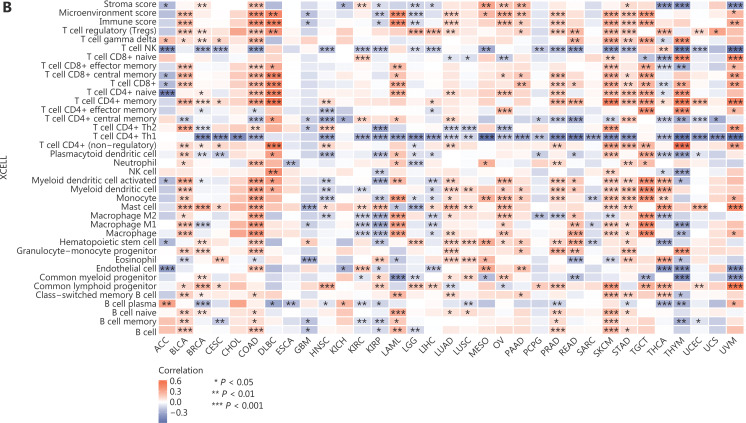
Immune-associated analysis and tumor mutational burden of BMAL1. (A) Tumor mutation burden: Spearman’s correlation analysis of TMB and BMAL1 gene expression. The transverse coordinates represent the correlation coefficient between genes and TMB, the ordinate coordinates represent different tumors, the circular point size represents the correlation coefficient, and different colors represent *P*-value aboriginality: bluer color in the diagram indicates smaller *P*-values. (B) The xCell immune infiltration score in multiple tumor tissues, and Spearman correlation analysis heatmap of BMAL1 (ARNTL) gene expression. The abscissa represents different tumor tissues, the ordinate represents different immune infiltration scores, different colors represent the correlation coefficient, negative values represent negative correlations, and positive values represent positive correlations. Stronger correlation is indicated by deeper color. *P < 0.05, **P < 0.01, **P < 0.001, with asterisks indicating significance. The dominance of the 2 sample groups passed the Wilcoxon test.

To better understand the role of circadian-mediated immunity in cancer progression, we performed a correlation analysis, and the results are depicted in **Figure 5A**. TMB reflects the density of the non-synonymous mutation distribution in protein-coding regions, usually represented by the total number of somatic tumor mutations in each MB tumor genome region. Tumors with high TMB may have more neoantigens that the immune system recognizes. As displayed in **Figure 5B**, in different cancers, BMAL1 has different or even opposite effects on the function of the immune system, in agreement with the contradictory points proposed above. Therefore, specific analyses should be performed for specific types of cancer.

## Conclusion and perspectives

**Figure 6:**
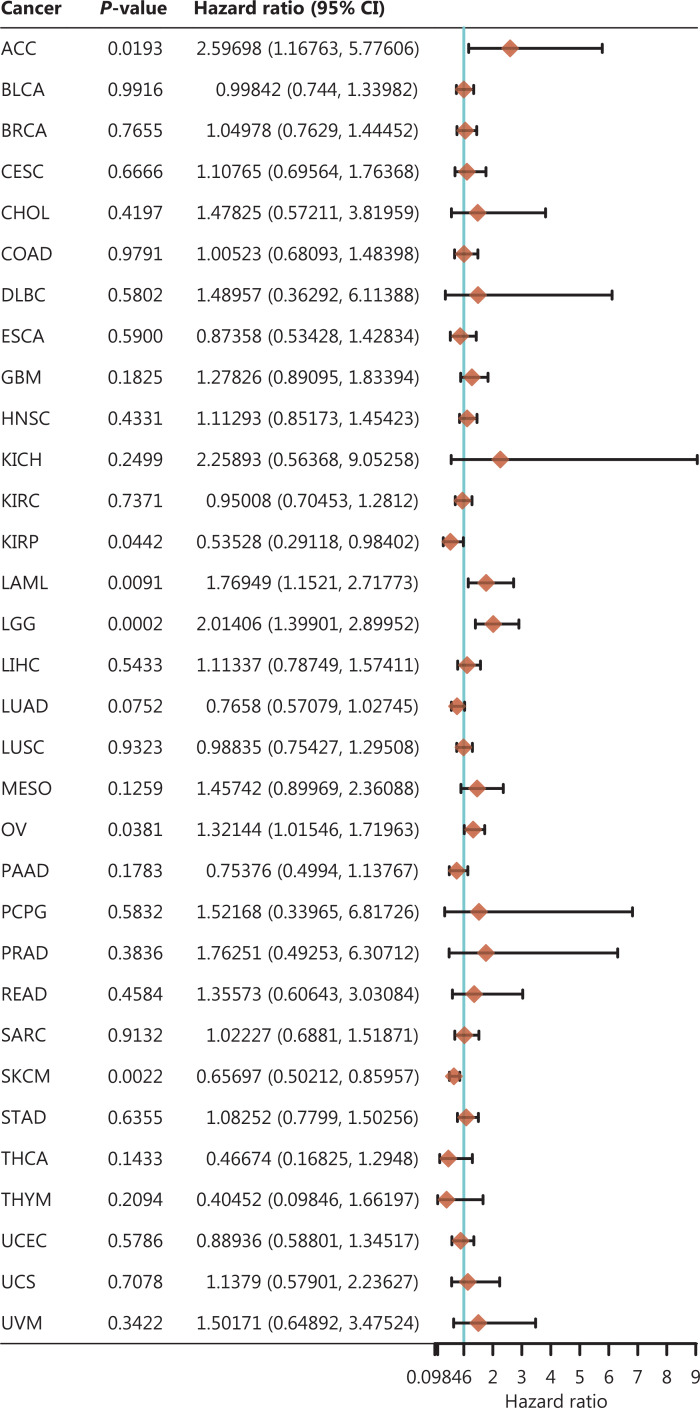
BMAL1 and prognosis of cancer. Forest plot: single-factor Cox analysis results for a single gene in multiple tumors, *P*-value, risk coefficient HR, and confidence interval. Abb: ACC, adrenocortical carcinoma; BLCA, bladder urothelial carcinoma; BRCA, breast invasive carcinoma; CESC, cervical squamous cell carcinoma and endocervical adenocarcinoma; CHOL, cholangiocarcinoma; COAD, colon adenocarcinoma; DLBC, lymphoid neoplasm diffuse large B-cell lymphoma; ESCA, esophageal carcinoma; GBM, glioblastoma multiforme; HNSC, head and neck squamous cell carcinoma; KICH, kidney chromophobe; KIRC, kidney renal clear cell carcinoma; KIRP, kidney renal papillary cell carcinoma; LAML, acute myeloid leukemia; LGG, brain lower grade glioma; LIHC, liver hepatocellular carcinoma; LUAD, lung adenocarcinoma; LUSC, lung squamous cell carcinoma; MESO, mesothelioma; OV, ovarian serous cystadenocarcinoma; PAAD, pancreatic adenocarcinoma; PCPG, pheochromocytoma and paraganglioma; PRAD, prostate adenocarcinoma; PEAD, rectal adenocarcinoma; SARC, sarcoma; SKCM, skin cutaneous melanoma; STAD, stomach adenocarcinoma; TGCT, testicular germ cell tumors; THCA, thyroid carcinoma; THYM, thymoma; UCEC, uterine corpus endometrial carcinoma; UCS, uterine carcinosarcoma; UVM, uveal melanoma.

Circadian rhythms, a physiological phenomenon widely present in various organisms in nature, have attracted the attention of various scientific researchers in recent decades, and are involved in the occurrence and development of various diseases to varying degrees. This article reviewed the roles of circadian rhythms in tumor diseases, including their composition, primary mechanisms, relationships with different tumors, and potential therapeutic targets. CLOCK and BMAL1 regulate various physiological processes in tumor cells, including tumor occurrence, tumor stem cell generation, apoptosis, and the endoplasmic reticulum stress response (EIF2DNA). On the basis of TTFL and second-order rhythm, the circadian clock is usually relatively stable *in vivo*. When this rhythm is broken, the occurrence and development of tumors are affected to varying degrees. Depending on whether BMAL1 expression has a positive or negative effect on tumor growth, different characteristics are observed in different tumors. Contradictions remain in the current research on the underlying mechanisms. However, the circadian clock as a target is not affected by the lack of mechanistic uncertainty, and this field has great research value in oncology, for example to provide prognostic indicators for cancer progression (**Figure 6**). This article summarized many possible effective targets for tumor therapy and provides a reference for related research. Further modification of small-molecule compounds known to regulate the biological clock, optimization of their pharmacokinetic properties, and minimization of adverse reactions are important research directions in this field. At present, research on most small molecule regulators remains in basic experimental stages, and biological clock regulating compounds with good therapeutic effects and pharmaceutical characteristics in animal models must enter the next stage of clinical trials. In addition, owing to the rhythmic expression of circadian clock genes, the optimal administration times for clock-targeted drugs and chemoradiotherapy are considered to maximize anti-tumor effects and minimize toxic and adverse effects. For example, treating cancer by administering mTOR inhibitors as at the peak of mTOR expression improves the survival rate in mice^232,233^. These unclear mechanisms are likely to gradually be elucidated in the future, thus deepening understanding of the role of CLOCK and BMAL1, and providing new ways to treat cancer.

## References

[1] Shafi AA, Knudsen KE (2019). Cancer and the circadian clock. Cancer Res.

[2] Ouyang Y, Andersson CR, Kondo T, Golden SS, Johnson CH (1998). Resonating circadian clocks enhance fitness in cyanobacteria. Proc Natl Acad Sci U S A.

[3] Turek FW (2016). Circadian clocks: not your grandfather’s clock. Science.

[4] Mohawk JA, Green CB, Takahashi JS (2012). Central and peripheral circadian clocks in mammals. Annu Rev Neurosci.

[5] Sancar A, van Gelder RN (2021). Clocks, cancer, and chronochemotherapy. Science.

[6] Partch CL, Green CB, Takahashi JS (2014). Molecular architecture of the mammalian circadian clock. Trends Cell Biol.

[7] Patke A, Young MW, Axelrod S (2020). Molecular mechanisms and physiological importance of circadian rhythms. Nat Rev Mol Cell Biol.

[8] Reppert SM, Weaver DR (2002). Coordination of circadian timing in mammals. Nature.

[9] Hastings MH, Reddy AB, Maywood ES (2003). A clockwork web: circadian timing in brain and periphery, in health and disease. Nat Rev Neurosci.

[10] Cederroth CR, Albrecht U, Bass J, Brown SA, Dyhrfjeld-Johnsen J, Gachon F (2019). Medicine in the fourth dimension. Cell Metab.

[11] Lubov JE, Cvammen W, Kemp MG (2021). The impact of the circadian clock on skin physiology and cancer development. Int J Mol Sci.

[12] Kume K, Zylka MJ, Sriram S, Shearman LP, Weaver DR, Jin X (1999). mCRY1 and mCRY2 are essential components of the negative limb of the circadian clock feedback loop. Cell.

[13] Griffin EA, Staknis D, Weitz CJ (1999). Light-independent role of CRY1 and CRY2 in the mammalian circadian clock. Science.

[14] Sangoram AM, Saez L, Antoch MP, Gekakis N, Staknis D, Whiteley A (1998). Mammalian circadian autoregulatory loop: a timeless ortholog and mPer1 interact and negatively regulate CLOCK-BMAL1-induced transcription. Neuron.

[15] Narasimamurthy R, Virshup DM (2021). The phosphorylation switch that regulates ticking of the circadian clock. Mol Cell.

[16] Kondratov RV, Chernov MV, Kondratova AA, Gorbacheva VY, Gudkov AV, Antoch MP (2003). BMAL1-dependent circadian oscillation of nuclear CLOCK: posttranslational events induced by dimerization of transcriptional activators of the mammalian clock system. Genes Dev.

[17] Kondratov RV, Shamanna RK, Kondratova AA, Gorbacheva VY, Antoch MP (2006). Dual role of the CLOCK/BMAL1 circadian complex in transcriptional regulation. FASEB J.

[18] Xu Y, Padiath QS, Shapiro RE, Jones CR, Wu SC, Saigoh N (2005). Functional consequences of a CKIdelta mutation causing familial advanced sleep phase syndrome. Nature.

[19] Etchegaray JP, Machida KK, Noton E, Constance CM, Dallmann R, Di Napoli MN (2009). Casein kinase 1 delta regulates the pace of the mammalian circadian clock. Mol Cell Biol.

[20] Philpott JM, Narasimamurthy R, Ricci CG, Freeberg AM, Hunt SR, Yee LE (2020). Casein kinase 1 dynamics underlie substrate selectivity and the PER2 circadian phosphoswitch. Elife.

[21] Green CB (2018). Circadian posttranscriptional regulatory mechanisms in mammals. Cold Spring Harb Perspect Biol.

[22] Shilts J, Chen G, Hughey JJ (2018). Evidence for widespread dysregulation of circadian clock progression in human cancer. PeerJ.

[23] Shostak A, Ruppert B, Ha N, Bruns P, Toprak UH, Eils R (2016). MYC/MIZ1-dependent gene repression inversely coordinates the circadian clock with cell cycle and proliferation. Nat Commun.

[24] Huber AL, Papp SJ, Chan AB, Henriksson E, Jordan SD, Kriebs A (2016). CRY2 and FBXL3 cooperatively degrade c-MYC. Mol Cell.

[25] Aviram R, Manella G, Kopelman N, Neufeld-Cohen A, Zwighaft Z, Elimelech M (2016). Lipidomics analyses reveal temporal and spatial lipid organization and uncover daily oscillations in intracellular organelles. Mol Cell.

[26] Blask DE, Dauchy RT, Dauchy EM, Mao L, Hill SM, Greene MW (2014). Light exposure at night disrupts host/cancer circadian regulatory dynamics: impact on the Warburg effect, lipid signaling and tumor growth prevention. PLoS One.

[27] Gamble KL, Berry R, Frank SJ, Young ME (2014). Circadian clock control of endocrine factors. Nat Rev Endocrinol.

[28] Greene MW (2012). Circadian rhythms and tumor growth. Cancer Lett.

[29] He W, Holtkamp S, Hergenhan SM, Kraus K, de Juan A, Weber J (2018). Circadian expression of migratory factors establishes lineage-specific signatures that guide the homing of leukocyte subsets to tissues. Immunity.

[30] Aiello I, Fedele MLM, Román F, Marpegan L, Caldart C, Chiesa JJ (2020). Circadian disruption promotes tumor-immune microenvironment remodeling favoring tumor cell proliferation. Sci Adv.

[31] Hadadi E, Taylor W, Li XM, Aslan Y, Villote M, Rivière J (2020). Chronic circadian disruption modulates breast cancer stemness and immune microenvironment to drive metastasis in mice. Nat Commun.

[32] Wu Y, Tao B, Zhang T, Fan Y, Mao R (2019). Pan-cancer analysis reveals disrupted circadian clock associates with T cell exhaustion. Front Immunol.

[33] Thorsson V, Gibbs DL, Brown SD, Wolf D, Bortone DS, Ou Yang TH (2018). The immune landscape of cancer. Immunity.

[34] Xie Y, Tang Q, Chen G, Xie M, Yu S, Zhao J (2019). New insights into the circadian rhythm and its related diseases. Front Physiol.

[35] Leng Y, Musiek ES, Hu K, Cappuccio FP, Yaffe K (2019). Association between circadian rhythms and neurodegenerative diseases. Lancet. Neurol.

[36] Kinouchi K, Sassone-Corsi P (2020). Metabolic rivalry: circadian homeostasis and tumorigenesis. Nat Rev Cancer.

[37] Schibler U, Gotic I, Saini C, Gos P, Curie T, Emmenegger Y (2015). Clock-talk: interactions between central and peripheral circadian oscillators in mammals. Cold Spring Harb Symp Quant Biol.

[38] Cox KH, Takahashi JS (2019). Circadian clock genes and the transcriptional architecture of the clock mechanism. J Mol Endocrinol.

[39] Finger AM, Dibner C, Kramer A (2020). Coupled network of the circadian clocks: a driving force of rhythmic physiology. FEBS Lett.

[40] Numata M, Hirano A, Yamamoto Y, Yasuda M, Miura N, Sayama K (2021). Metastasis of breast cancer promoted by circadian rhythm disruption due to light/dark shift and its prevention by dietary quercetin in mice. J Circadian Rhythms.

[41] Ha NH, Long J, Cai Q, Shu XO, Hunter KW (2016). The circadian rhythm gene Arntl2 is a metastasis susceptibility gene for estrogen receptor-negative breast cancer. PLoS Genetics.

[42] Chen J, Liu A, Lin Z, Wang B, Chai X, Chen S (2020). Downregulation of the circadian rhythm regulator HLF promotes multiple-organ distant metastases in non-small cell lung cancer through PPAR/NF-κb signaling. Cancer Lett.

[43] Wang Y, Sun N, Lu C, Bei Y, Qian R, Hua L (2017). Upregulation of circadian gene ‘hClock’ contribution to metastasis of colorectal cancer. Int J Oncol.

[44] Koritala BSC, Porter KI, Arshad OA, Gajula RP, Mitchell HD, Arman T (2021). Night shift schedule causes circadian dysregulation of DNA repair genes and elevated DNA damage in humans. J Pineal Res.

[45] Dun A, Zhao X, Jin X, Wei T, Gao X, Wang Y (2020). Association between night-shift work and cancer risk: updated systematic review and meta-analysis. Front Oncol.

[46] Srour B, Plancoulaine S, Andreeva VA, Fassier P, Julia C, Galan P (2018). Circadian nutritional behaviours and cancer risk: new insights from the NutriNet-santé prospective cohort study: disclaimers. Int J Cancer.

[47] Lou X, Wang H, Tu Y, Tan W, Jiang C, Sun J (2021). Alterations of sleep quality and circadian rhythm genes expression in elderly thyroid nodule patients and risks associated with thyroid malignancy. Sci Rep.

[48] Kondratov RV, Kondratova AA, Gorbacheva VY, Vykhovanets OV, Antoch MP (2006). Early aging and age-related pathologies in mice deficient in BMAL1, the core componentof the circadian clock. Genes Dev.

[49] Yu EA, Weaver DR (2011). Disrupting the circadian clock: gene-specific effects on aging, cancer, and other phenotypes. Aging.

[50] Papagiannakopoulos T, Bauer MR, Davidson SM, Heimann M, Subbaraj L, Bhutkar A (2016). Circadian rhythm disruption promotes lung tumorigenesis. Cell Metab.

[51] Sulli G, Lam MTY, Panda S (2019). Interplay between circadian clock and cancer: new frontiers for cancer treatment. Trends Cancer.

[52] Zhou L, Luo Z, Li Z, Huang Q (2020). Circadian clock is associated with tumor microenvironment in kidney renal clear cell carcinoma. Aging.

[53] Yang Y, Yuan G, Xie H, Wei T, Zhu D, Cui J (2019). Circadian clock associates with tumor microenvironment in thoracic cancers. Aging.

[54] Pavlova NN, Thompson CB (2016). The emerging hallmarks of cancer metabolism. Cell Metab.

[55] Chen WD, Wen MS, Shie SS, Lo YL, Wo HT, Wang CC (2014). The circadian rhythm controls telomeres and telomerase activity. Biochem Biophys Res Commun.

[56] Ruan W, Yuan X, Eltzschig HK (2021). Circadian rhythm as a therapeutic target. Nat Rev Drug Discov.

[57] Chen P, Hsu WH, Han J, Xia Y, DePinho RA (2021). Cancer stemness meets immunity: from mechanism to therapy. Cell Rep.

[58] Chen P, Hsu WH, Chang A, Tan Z, Lan Z, Zhou A (2020). Circadian regulator CLOCK recruits immune-suppressive microglia into the GBM tumor microenvironment. Cancer Discov.

[59] Dong Z, Zhang G, Qu M, Gimple RC, Wu Q, Qiu Z (2019). Targeting glioblastoma stem cells through disruption of the circadian clock. Cancer Discov.

[60] Puram RV, Kowalczyk MS, de Boer CG, Schneider RK, Miller PG, McConkey M (2016). Core circadian clock genes regulate leukemia stem cells in AML. Cell.

[61] Hu Z, Brooks SA, Dormoy V, Hsu CW, Hsu HY, Lin LT (2015). Assessing the carcinogenic potential of low-dose exposures to chemical mixtures in the environment: focus on the cancer hallmark of tumor angiogenesis. Carcinogenesis.

[62] Jiang X, Wang J, Deng X, Xiong F, Zhang S, Gong Z (2020). The role of microenvironment in tumor angiogenesis. J Exp Clin Cancer Res.

[63] Hanahan D, Weinberg RA (2011). Hallmarks of cancer: the next generation. Cell.

[64] Shalapour S, Karin M (2019). Pas de deux: control of anti-tumor immunity by cancer-associated inflammation. Immunity.

[65] Ramos CA, Ouyang C, Qi Y, Chung Y, Cheng CT, LaBarge MA (2020). A non-canonical function of BMAL1 metabolically limits obesity-promoted triple-negative breast cancer. iScience.

[66] Matsunaga N, Ogino T, Hara Y, Tanaka T, Koyanagi S, Ohdo S (2018). Optimized dosing schedule based on circadian dynamics of mouse breast cancer stem cells improves the antitumor effects of aldehyde dehydrogenase inhibitor. Cancer Res.

[67] Chefetz I, Grimley E, Yang K, Hong L, Vinogradova EV, Suciu R (2019). A pan-ALDH1A inhibitor induces necroptosis in ovarian cancer stem-like cells. Cell Rep.

[68] Kojima M, Hosoda H, Date Y, Nakazato M, Matsuo H, Kangawa K (1999). Ghrelin is a growth-hormone-releasing acylated peptide from stomach. Nature.

[69] Avau B, Carbone F, Tack J, Depoortere I (2013). Ghrelin signaling in the gut, its physiological properties, and therapeutic potential. Neurogastroenterol Motil.

[70] Müller TD, Nogueiras R, Andermann ML, Andrews ZB, Anker SD, Argente J (2015). Ghrelin. Mol Metab.

[71] Waseem T, Javaid Ur R, Ahmad F, Azam M, Qureshi MA (2008). Role of ghrelin axis in colorectal cancer: a novel association. Peptides.

[72] Murata M, Okimura Y, Iida K, Matsumoto M, Sowa H, Kaji H (2002). Ghrelin modulates the downstream molecules of insulin signaling in hepatoma cells. J Biol Chem.

[73] De Vriese C, Grégoire F, De Neef P, Robberecht P, Delporte C (2005). Ghrelin is produced by the human erythroleukemic HEL cell line and involved in an autocrine pathway leading to cell proliferation. Endocrinology.

[74] Fung JNT, Seim I, Wang D, Obermair A, Chopin LK, Chen C (2010). Expression and in vitro functions of the ghrelin axis in endometrial cancer. Horm Cancer.

[75] Duxbury MS, Waseem T, Ito H, Robinson MK, Zinner MJ, Ashley SW (2003). Ghrelin promotes pancreatic adenocarcinoma cellular proliferation and invasiveness. Biochem Biophys Res Commun.

[76] Lien GS, Lin CH, Yang YL, Wu MS, Chen BC (2016). Ghrelin induces colon cancer cell proliferation through the GHS-R, Ras, PI3K, Akt, and mTOR signaling pathways. Eur J Pharmacol.

[77] Kraus D, Reckenbeil J, Wenghoefer M, Stark H, Frentzen M, Allam JP (2016). Ghrelin promotes oral tumor cell proliferation by modifying GLUT1 expression. Cell Mol Life Sci.

[78] Díaz-Lezama N, Hernández-Elvira M, Sandoval A, Monroy A, Felix R, Monjaraz E (2010). Ghrelin inhibits proliferation and increases T-type Ca2+ channel expression in PC-3 human prostate carcinoma cells. Biochem Biophys Res Commun.

[79] Yeh AH, Jeffery PL, Duncan RP, Herington AC, Chopin LK (2005). Ghrelin and a novel preproghrelin isoform are highly expressed in prostate cancer and ghrelin activates mitogen-activated protein kinase in prostate cancer. Clin Cancer Res.

[80] Segers A, Desmet L, Sun S, Verbeke K, Tack J, Depoortere I (2020). Night-time feeding of Bmal1-/- mice restores SCFA rhythms and their effect on ghrelin. J Endocrinol.

[81] Cummings DE, Purnell JQ, Frayo RS, Schmidova K, Wisse BE, Weigle DS (2001). A preprandial rise in plasma ghrelin levels suggests a role in meal initiation in humans. Diabetes.

[82] Bodosi B, Gardi J, Hajdu I, Szentirmai E, Obal F, Krueger JM (2004). Rhythms of ghrelin, leptin, and sleep in rats: effects of the normal diurnal cycle, restricted feeding, and sleep deprivation. Am J Physiol Regul Integr Comp Physiol.

[83] Yildiz BO, Suchard MA, Wong ML, McCann SM, Licinio J (2004). Alterations in the dynamics of circulating ghrelin, adiponectin, and leptin in human obesity. Proc Natl Acad Sci U S A.

[84] Laermans J, Vancleef L, Tack J, Depoortere I (2015). Role of the clock gene Bmal1 and the gastric ghrelin-secreting cell in the circadian regulation of the ghrelin-GOAT system. Sci Rep.

[85] Diehl JA, Fuchs SY, Koumenis C (2011). The cell biology of the unfolded protein response. Gastroenterology.

[86] Bu Y, Yoshida A, Chitnis N, Altman BJ, Tameire F, Oran A (2018). A PERK-miR-211 axis suppresses circadian regulators and protein synthesis to promote cancer cell survival. Nat Cell Biol.

[87] Hart LS, Cunningham JT, Datta T, Dey S, Tameire F, Lehman SL (2012). ER stress-mediated autophagy promotes Myc-dependent transformation and tumor growth. J Clin Invest.

[88] Cramer T, Yamanishi Y, Clausen BE, Förster I, Pawlinski R, Mackman N (2003). HIF-1alpha is essential for myeloid cell-mediated inflammation. Cell.

[89] Masson N, Ratcliffe PJ (2014). Hypoxia signaling pathways in cancer metabolism: the importance of co-selecting interconnected physiological pathways. Cancer Metab.

[90] Semenza GL, Roth PH, Fang HM, Wang GL (1994). Transcriptional regulation of genes encoding glycolytic enzymes by hypoxia-inducible factor 1. J Biol Chem.

[91] Doedens AL, Stockmann C, Rubinstein MP, Liao D, Zhang N, DeNardo DG (2010). Macrophage expression of hypoxia-inducible factor-1 alpha suppresses T-cell function and promotes tumor progression. Cancer Res.

[92] Steggerda SM, Bennett MK, Chen J, Emberley E, Huang T, Janes JR (2017). Inhibition of arginase by CB-1158 blocks myeloid cell-mediated immune suppression in the tumor microenvironment. J Immunother Cancer.

[93] Nguyen KD, Fentress SJ, Qiu Y, Yun K, Cox JS, Chawla A (2013). Circadian gene Bmal1 regulates diurnal oscillations of Ly6C(hi) inflammatory monocytes. Science.

[94] Alexander RK, Liou YH, Knudsen NH, Starost KA, Xu C, Hyde AL (2020). Bmal1 integrates mitochondrial metabolism and macrophage activation. ELife.

[95] DeMarzo AM, Nelson WG, Isaacs WB, Epstein JI (2003). Pathological and molecular aspects of prostate cancer. Lancet (London, England).

[96] Albihn A, Johnsen JI, Henriksson MA (2010). MYC in oncogenesis and as a target for cancer therapies. Adv Cancer Res.

[97] Fernandez PC, Frank SR, Wang L, Schroeder M, Liu S, Greene J (2003). Genomic targets of the human c-Myc protein. Genes Dev.

[98] Adhikary S, Eilers M (2005). Transcriptional regulation and transformation by Myc proteins. Nat Rev Mol Cell Biol.

[99] Larsson LG, Henriksson MA (2010). The Yin and Yang functions of the Myc oncoprotein in cancer development and as targets for therapy. Exp Cell Res.

[100] Altman BJ, Hsieh AL, Sengupta A, Krishnanaiah SY, Stine ZE, Walton ZE (2015). MYC disrupts the circadian clock and metabolism in cancer cells. Cell Metab.

[101] Gery S, Komatsu N, Baldjyan L, Yu A, Koo D, Koeffler HP (2006). The circadian gene per1 plays an important role in cell growth and DNA damage control in human cancer cells. Mol Cell.

[102] Hua H, Wang Y, Wan C, Liu Y, Zhu B, Yang C (2006). Circadian gene mPer2 overexpression induces cancer cell apoptosis. Cancer Sci.

[103] Yang X, Wood PA, Ansell CM, Quiton DFT, Oh EY, Du-Quiton J (2009). The circadian clock gene Per1 suppresses cancer cell proliferation and tumor growth at specific times of day. Chronobiol Int.

[104] Repouskou A, Prombona A (2016). c-MYC targets the central oscillator gene Per1 and is regulated by the circadian clock at the post-transcriptional level. Biochim Biophys Acta.

[105] O’Connor K, Chen M (2013). Dynamic functions of RhoA in tumor cell migration and invasion. Small GTPases.

[106] Chiba S, Enami T, Ogawa S, Sakata-Yanagimoto M (2015). G17V RHOA: Genetic evidence of GTP-unbound RHOA playing a role in tumorigenesis in T cells. Small GTPases.

[107] Nomikou E, Stournaras C, Kardassis D (2017). Functional analysis of the promoters of the small GTPases RhoA and RhoB in embryonic stem cells. Biochem Biophys Res Commun.

[108] Ma TJ, Zhang ZW, Lu YL, Zhang YY, Tao DC, Liu YQ (2018). CLOCK and BMAL1 stabilize and activate RHOA to promote F-actin formation in cancer cells. Exp Mol Med.

[109] Ramanathan C, Kathale ND, Liu D, Lee C, Freeman DA, Hogenesch JB (2018). mTOR signaling regulates central and peripheral circadian clock function. PLoS Genet.

[110] Memmott RM, Dennis PA (2009). Akt-dependent and -independent mechanisms of mTOR regulation in cancer. Cell Signal.

[111] Chiang GG, Abraham RT (2007). Targeting the mTOR signaling network in cancer. Trends Mol Med.

[112] Alzahrani AS (2019). PI3K/Akt/mTOR inhibitors in cancer: at the bench and bedside. Semin Cancer Biol.

[113] Hardie DG (2007). AMP-activated/SNF1 protein kinases: conserved guardians of cellular energy. Nat Rev Mol Cell Biol.

[114] Lee Y, Kim EK (2013). AMP-activated protein kinase as a key molecular link between metabolism and clockwork. Exp Mol Med.

[115] Um JH, Yang S, Yamazaki S, Kang H, Viollet B, Foretz M (2007). Activation of 5’-AMP-activated kinase with diabetes drug metformin induces casein kinase Iepsilon (CKIepsilon)-dependent degradation of clock protein mPer2. J Biol Chem.

[116] Liang J, Mills GB (2013). AMPK: a contextual oncogene or tumor suppressor?. Cancer Res.

[117] Carling D (2017). AMPK signalling in health and disease. Curr Opin Cell Biol.

[118] Li Q, Xia D, Wang Z, Liu B, Zhang J, Peng P (2021). Circadian rhythm gene PER3 negatively regulates stemness of prostate cancer stem cells via WNT/β-catenin signaling in tumor microenvironment. Front Cell Dev Biol.

[119] Maiese K (2017). Moving to the rhythm with clock (Circadian) genes, autophagy, mTOR, and SIRT1 in degenerative disease and cancer. Curr Neurovasc Res.

[120] Okazaki F, Matsunaga N, Okazaki H, Azuma H, Hamamura K, Tsuruta A (2016). Circadian clock in a mouse colon tumor regulates intracellular iron levels to promote tumor progression. J Biol Chem.

[121] Shen Y, Endale M, Wang W, Morris AR, Francey LJ, Harold RL (2021). NF-κB modifies the mammalian circadian clock through interaction with the core clock protein BMAL1. PLoS Genet.

[122] Kubra S, Zhang H, Si Y, Gao X, Wang T, Pan L (2021). REGγ regulates circadian clock by modulating BMAL1 protein stability. Cell Death Discov.

[123] Moreno-Smith M, Milazzo G, Tao L, Fekry B, Zhu B, Mohammad MA (2021). Restoration of the molecular clock is tumor suppressive in neuroblastoma. Nat Commun.

[124] Jiang W, Zhao S, Jiang X, Zhang E, Hu G, Hu B (2016). The circadian clock gene Bmal1 acts as a potential anti-oncogene in pancreatic cancer by activating the p53 tumor suppressor pathway. Cancer Lett.

[125] Liu L, Liao JZ, He XX, Li PY (2017). The role of autophagy in hepatocellular carcinoma: friend or foe. Oncotarget.

[126] Torre LA, Bray F, Siegel RL, Ferlay J, Lortet-Tieulent J, Jemal A (2015). Global cancer statistics, 2012. CA Cancer J Clin.

[127] (2020). World Health Organization, Projections of Mortality and Causes of Death, 2016 to 2060.

[128] Singal AG, El-Serag HB (2015). Hepatocellular carcinoma from epidemiology to prevention: translating knowledge into practice. Clin Gastroenterol Hepatol.

[129] Fattovich G, Stroffolini T, Zagni I, Donato F (2004). Hepatocellular carcinoma in cirrhosis: incidence and risk factors. Gastroenterology.

[130] Molina-Aguilar C, Guerrero-Carrillo MdJ, Espinosa-Aguirre JJ, Olguin-Reyes S, Castro-Belio T, Vázquez-Martínez O (2017). Time-caloric restriction inhibits the neoplastic transformation of cirrhotic liver in rats treated with diethylnitrosamine. Carcinogenesis.

[131] Krizkova S, Kepinska M, Emri G, Rodrigo MAM, Tmejova K, Nerudova D (2016). Microarray analysis of metallothioneins in human diseases--A review. J Pharm Biomed Anal.

[132] Fujie T, Segawa Y, Yoshida E, Kimura T, Fujiwara Y, Yamamoto C (2016). Induction of metallothionein isoforms by copper diethyldithiocarbamate in cultured vascular endothelial cells. J Toxicol Sci.

[133] Klaassen CD, Liu J, Choudhuri S (1999). Metallothionein: an intracellular protein to protect against cadmium toxicity. Annu Rev Pharmacol Toxicol.

[134] Li H, Lu YF, Chen H, Liu J (2017). Dysregulation of metallothionein and circadian genes in human hepatocellular carcinoma. Chronobiol Int.

[135] Hill SM, Belancio VP, Dauchy RT, Xiang S, Brimer S, Mao L (2015). Melatonin: an inhibitor of breast cancer. Endocr Relat Cancer.

[136] Reiter RJ, Rosales-Corral SA, Tan DX, Acuna-Castroviejo D, Qin L, Yang SF (2017). Melatonin, a full service anti-cancer agent: inhibition of initiation, progression and metastasis. Int J Mol Sci.

[137] Li Y, Li S, Zhou Y, Meng X, Zhang JJ, Xu DP (2017). Melatonin for the prevention and treatment of cancer. Oncotarget.

[138] Sánchez DI, González-Fernández B, Crespo I, San-Miguel B, Álvarez M, González-Gallego J (2018). Melatonin modulates dysregulated circadian clocks in mice with diethylnitrosamine-induced hepatocellular carcinoma. J Pineal Res.

[139] Sladek FM, Zhong WM, Lai E, Darnell JE (1990). Liver-enriched transcription factor HNF-4 is a novel member of the steroid hormone receptor superfamily. Genes Dev.

[140] Battle MA, Konopka G, Parviz F, Gaggl AL, Yang C, Sladek FM (2006). Hepatocyte nuclear factor 4alpha orchestrates expression of cell adhesion proteins during the epithelial transformation of the developing liver. Proc Natl Acad Sci U S A.

[141] Bonzo JA, Ferry CH, Matsubara T, Kim JH, Gonzalez FJ (2012). Suppression of hepatocyte proliferation by hepatocyte nuclear factor 4α in adult mice. J Biol Chem.

[142] Chellappa K, Deol P, Evans JR, Vuong LM, Chen G, Briançon N (2016). Opposing roles of nuclear receptor HNF4α isoforms in colitis and colitis-associated colon cancer. ELife.

[143] Walesky C, Apte U (2015). Role of hepatocyte nuclear factor 4α (HNF4α) in cell proliferation and cancer. Gene Expr.

[144] Vuong LM, Chellappa K, Dhahbi JM, Deans JR, Fang B, Bolotin E (2015). Differential effects of hepatocyte nuclear factor 4α isoforms on tumor growth and T-cell factor 4/AP-1 interactions in human colorectal cancer cells. Mol Cell Biol.

[145] Ning BF, Ding J, Yin C, Zhong W, Wu K, Zeng X (2010). Hepatocyte nuclear factor 4 alpha suppresses the development of hepatocellular carcinoma. Cancer Res.

[146] Hatziapostolou M, Polytarchou C, Aggelidou E, Drakaki A, Poultsides GA, Jaeger SA (2011). An HNF4α-miRNA inflammatory feedback circuit regulates hepatocellular oncogenesis. Cell.

[147] Walesky C, Edwards G, Borude P, Gunewardena S, O’Neil M, Yoo B (2013). Hepatocyte nuclear factor 4 alpha deletion promotes diethylnitrosamine-induced hepatocellular carcinoma in rodents. Hepatology.

[148] Tanaka T, Jiang S, Hotta H, Takano K, Iwanari H, Sumi K (2006). Dysregulated expression of P1 and P2 promoter-driven hepatocyte nuclear factor-4alpha in the pathogenesis of human cancer. J Pathol.

[149] Chellappa K, Jankova L, Schnabl JM, Pan S, Brelivet Y, Fung CLS (2012). Src tyrosine kinase phosphorylation of nuclear receptor HNF4α correlates with isoform-specific loss of HNF4α in human colon cancer. Proc Natl Acad Sci U S A.

[150] Fekry B, Ribas-Latre A, Baumgartner C, Deans JR, Kwok C, Patel P (2018). Incompatibility of the circadian protein BMAL1 and HNF4α in hepatocellular carcinoma. Nat Commun.

[151] Biller LH, Schrag D (2021). Diagnosis and treatment of metastatic colorectal cancer: a review. JAMA.

[152] Sung H, Ferlay J, Siegel RL, Laversanne M, Soerjomataram I, Jemal A (2021). Global cancer statistics 2020: GLOBOCAN estimates of incidence and mortality worldwide for 36 cancers in 185 countries. CA Cancer J Clin.

[153] Siegel RL, Medhanie GA, Fedewa SA, Jemal A (2019). State variation in early-onset colorectal cancer in the United States, 1995-2015. J Natl Cancer Inst.

[154] Zhang Y, Devocelle A, Souza L, Foudi A, Tenreira Bento S, Desterke C (2020). BMAL1 knockdown triggers different colon carcinoma cell fates by altering the delicate equilibrium between AKT/mTOR and P53/P21 pathways. Aging.

[155] Stokes K, Nunes M, Trombley C, Flôres DEFL, Wu G, Taleb Z (2021). The circadian clock gene, Bmal1, regulates intestinal stem cell signaling and represses tumor initiation. Cell Mol Gastroenterol Hepatol.

[156] Liu JL, Wang CY, Cheng TY, Rixiati Y, Ji C, Deng M (2021). Circadian clock disruption suppresses PDL1 intraepithelial B cells in experimental colitis and colitis-associated colorectal cancer. Cell Mol Gastroenterol Hepatol.

[157] Zhang Y, Ma J, Zhang S, Deng G, Wu X, He J (2015). A prognostic analysis of 895 cases of stage III colon cancer in different colon subsites. Int J Colorectal Dis.

[158] Huisman SA, Oklejewicz M, Ahmadi AR, Tamanini F, Ijzermans JNM, van der Horst GTJ (2015). Colorectal liver metastases with a disrupted circadian rhythm phase shift the peripheral clock in liver and kidney. Int J Cancer.

[159] Dong P, Wang Y, Liu Y, Zhu C, Lin J, Qian R (2022). BMAL1 induces colorectal cancer metastasis by stimulating exosome secretion. Mol Biol Rep.

[160] Siegel RL, Miller KD, Jemal A (2018). Cancer statistics, 2018. CA Cancer J Clin.

[161] Dai X, Li T, Bai Z, Yang Y, Liu X, Zhan J (2015). Breast cancer intrinsic subtype classification, clinical use and future trends. Am J Cancer Res.

[162] Xiang S, Mao L, Duplessis T, Yuan L, Dauchy R, Dauchy E (2012). Oscillation of clock and clock controlled genes induced by serum shock in human breast epithelial and breast cancer cells: regulation by melatonin. Breast Cancer (Auckl).

[163] Rossetti S, Esposito J, Corlazzoli F, Gregorski A, Sacchi N (2012). Entrainment of breast (cancer) epithelial cells detects distinct circadian oscillation patterns for clock and hormone receptor genes. Cell Cycle.

[164] Lesicka M, Jabłońska E, Wieczorek E, Seroczyńska B, Siekierzycka A, Skokowski J (2018). Altered circadian genes expression in breast cancer tissue according to the clinical characteristics. PLoS One.

[165] Broadberry E, McConnell J, Williams J, Yang N, Zindy E, Leek A (2018). Disrupted circadian clocks and altered tissue mechanics in primary human breast tumours. Breast Cancer Res.

[166] Korkmaz T, Aygenli F, Emisoglu H, Ozcelik G, Canturk A, Yilmaz S (2018). Opposite carcinogenic effects of circadian clock gene BMAL1. Sci Rep.

[167] Kwon YJ, Seo EB, Kwon SH, Lee SH, Kim SK, Park SK (2020). Extracellular Acidosis Promotes Metastatic Potency via Decrease of the BMAL1 Circadian Clock Gene in Breast Cancer. Cells.

[168] Sharma D, Kumar S, Narasimhan B (2018). Estrogen alpha receptor antagonists for the treatment of breast cancer: a review. Chem Cent J.

[169] Xie F, Wang L, Liu Y, Liu Z, Zhang Z, Pei J (2020). ASMT regulates tumor metastasis through the circadian clock system in triple-negative breast cancer. Front Oncol.

[170] Boese AC, Kang S (2020). Tumor progression of breast cancer during hyperinsulinemic obesity. Trends Mol Med.

[171] Hambardzumyan D, Gutmann DH, Kettenmann H (2016). The role of microglia and macrophages in glioma maintenance and progression. Nat Neurosci.

[172] Mantovani A, Sica A, Sozzani S, Allavena P, Vecchi A, Locati M (2004). The chemokine system in diverse forms of macrophage activation and polarization. Trends Immunol.

[173] Verreck FAW, de Boer T, Langenberg DML, Hoeve MA, Kramer M, Vaisberg E (2004). Human IL-23-producing type 1 macrophages promote but IL-10-producing type 2 macrophages subvert immunity to (myco)bacteria. Proc Natl Acad Sci U S A.

[174] Qin C, Zhou LQ, Ma XT, Hu ZW, Yang S, Chen M (2019). Dual functions of microglia in ischemic stroke. Neurosci Bull.

[175] Li X, Guan J, Jiang Z, Cheng S, Hou W, Yao J (2021). Microglial exosome miR-7239-3p promotes glioma progression by regulating circadian genes. Neurosci Bull.

[176] de Assis LVM, Mendes D, Silva MM, Kinker GS, Pereira-Lima I, Moraes MN (2020). Melanopsin mediates UVA-dependent modulation of proliferation, pigmentation, apoptosis, and molecular clock in normal and malignant melanocytes. Biochim Biophys Acta Mol Cell Res.

[177] de Assis LVM, Moraes MN, da Silveira Cruz-Machado S, Castrucci AML (2016). The effect of white light on normal and malignant murine melanocytes: a link between opsins, clock genes, and melanogenesis. Biochim Biophys Acta.

[178] Zhanfeng N, Yanhui L, Zhou F, Shaocai H, Guangxing L, Hechun X (2015). Circadian genes Per1 and Per2 increase radiosensitivity of glioma in vivo. Oncotarget.

[179] Shen H, Cook K, Gee HE, Hau E (2020). Hypoxia, metabolism, and the circadian clock: new links to overcome radiation resistance in high-grade gliomas. J Exp Clin Cancer Res CR.

[180] Wagner PM, Prucca CG, Velazquez FN, Sosa Alderete LG, Caputto BL, Guido ME (2021). Temporal regulation of tumor growth in nocturnal mammals: in vivo studies and chemotherapeutical potential. FASEB J.

[181] Katamune C, Koyanagi S, Hashikawa KI, Kusunose N, Akamine T, Matsunaga N (2019). Mutation of the gene encoding the circadian clock component PERIOD2 in oncogenic cells confers chemoresistance by up-regulating the Aldh3a1 gene. J Biol Chem.

[182] Ferrara N, Hillan KJ, Gerber HP, Novotny W (2004). Discovery and development of bevacizumab, an anti-VEGF antibody for treating cancer. Nat Rev Drug Discov.

[183] De Palma M, Biziato D, Petrova TV (2017). Microenvironmental regulation of tumour angiogenesis. Nat Rev Cancer.

[184] Koyanagi S, Kuramoto Y, Nakagawa H, Aramaki H, Ohdo S, Soeda S (2003). A molecular mechanism regulating circadian expression of vascular endothelial growth factor in tumor cells. Cancer Res.

[185] Peek CB, Levine DC, Cedernaes J, Taguchi A, Kobayashi Y, Tsai SJ (2017). Circadian clock interaction with HIF1α mediates oxygenic metabolism and anaerobic glycolysis in skeletal muscle. Cell Metabolism.

[186] Ma Z, Jin X, Qian Z, Li F, Xu M, Zhang Y (2019). Deletion of clock gene Bmal1 impaired the chondrocyte function due to disruption of the HIF1α-VEGF signaling pathway. Cell Cycle.

[187] Burgermeister E, Battaglin F, Eladly F, Wu W, Herweck F, Schulte N (2019). Aryl hydrocarbon receptor nuclear translocator-like (ARNTL/BMAL1) is associated with bevacizumab resistance in colorectal cancer via regulation of vascular endothelial growth factor A. EBioMedicine.

[188] Thiery JP, Acloque H, Huang RYJ, Nieto MA (2009). Epithelial-mesenchymal transitions in development and disease. Cell.

[189] Zhang J, Tian XJ, Xing J (2016). Signal transduction pathways of EMT induced by TGF-β, SHH, and WNT and their crosstalks. J Clin Med.

[190] Jeanes A, Gottardi CJ, Yap AS (2008). Cadherins and cancer: how does cadherin dysfunction promote tumor progression?. Oncogene.

[191] Zhang Y, Devocelle A, Desterke C, de Souza LEB, Hadadi É, Acloque H (2021). BMAL1 knockdown leans epithelial-mesenchymal balance toward epithelial properties and decreases the chemoresistance of colon carcinoma cells. Int J Mol Sci.

[192] Slat EA, Sponagel J, Marpegan L, Simon T, Kfoury N, Kim A (2017). Cell-intrinsic, Bmal1-dependent circadian regulation of temozolomide sensitivity in glioblastoma. J Biol Rhythms.

[193] Wagner PM, Sosa Alderete LG, Gorné LD, Gaveglio V, Salvador G, Pasquaré S (2019). Proliferative glioblastoma cancer cells exhibit persisting temporal control of metabolism and display differential temporal drug susceptibility in chemotherapy. Mol Neurobiol.

[194] Jiang W, Zhao S, Shen J, Guo L, Sun Y, Zhu Y (2018). The MiR-135b-BMAL1-YY1 loop disturbs pancreatic clockwork to promote tumourigenesis and chemoresistance. Cell Death Dis.

[195] Tang Q, Cheng B, Xie M, Chen Y, Zhao J, Zhou X (2017). Circadian clock gene Bmal1 inhibits tumorigenesis and increases paclitaxel sensitivity in tongue squamous cell carcinoma. Cancer Res.

[196] Trebucq LL, Cardama GA, Lorenzano Menna P, Golombek DA, Chiesa JJ, Marpegan L (2021). Timing of novel drug 1A-116 to circadian rhythms improves therapeutic effects against glioblastoma. Pharmaceutics.

[197] Cardama GA, Gonzalez N, Ciarlantini M, Gandolfi Donadío L, Comin MJ, Alonso DF (2014). Proapoptotic and antiinvasive activity of Rac1 small molecule inhibitors on malignant glioma cells. OncoTargets and Therapy.

[198] Cabrera M, Echeverria E, Lenicov FR, Cardama G, Gonzalez N, Davio C (2017). Pharmacological Rac1 inhibitors with selective apoptotic activity in human acute leukemic cell lines. Oncotarget.

[199] Parri M, Chiarugi P (2010). Rac and Rho GTPases in cancer cell motility control. Cell Commun Signal.

[200] Ridley AJ (2015). Rho GTPase signalling in cell migration. Curr Opin Cell Biol.

[201] Teixeira AAS, Biondo LA, Silveira LS, Lima EA, Batatinha HA, Diniz TA (2020). Doxorubicin modulated clock genes and cytokines in macrophages extracted from tumor-bearing mice. Cancer Biol Ther.

[202] Mendes MCS, Pimentel GD, Costa FO, Carvalheira JBC (2015). Molecular and neuroendocrine mechanisms of cancer cachexia. J Endocrinol.

[203] Kinsey E, Ajazi E, Wang X, Johnston MAM, Crawford J (2018). Predictors of physical and functional loss in advanced-stage lung cancer patients receiving platinum chemotherapy. J Thorac Oncol.

[204] Muliawati Y, Haroen H, Rotty LWA (2012). Cancer anorexia - cachexia syndrome. Acta Med Indones.

[205] von Haehling S, Anker SD (2012). Cachexia as major underestimated unmet medical need: facts and numbers. Int J Cardiol.

[206] Colldén G, Tschöp MH, Müller TD (2017). Therapeutic potential of targeting the ghrelin pathway. Int J Mol Sci.

[207] Chen JA, Splenser A, Guillory B, Luo J, Mendiratta M, Belinova B (2015). Ghrelin prevents tumour- and cisplatin-induced muscle wasting: characterization of multiple mechanisms involved. J Cachexia Sarcopenia Muscle.

[208] Hatanaka M, Konishi M, Ishida J, Saito M, Springer J (2015). Novel mechanism of ghrelin therapy for cachexia. J Cachexia Sarcopenia Muscle.

[209] Lundholm K, Gunnebo L, Körner U, Iresjö BM, Engström C, Hyltander A (2010). Effects by daily long term provision of ghrelin to unselected weight-losing cancer patients: a randomized double-blind study. Cancer.

[210] Doruk YU, Yarparvar D, Akyel YK, Gul S, Taskin AC, Yilmaz F (2020). A CLOCK-binding small molecule disrupts the interaction between CLOCK and BMAL1 and enhances circadian rhythm amplitude. J Biol Chem.

[211] Saxton RA, Sabatini DM (2017). mTOR signaling in growth, metabolism, and disease. Cell.

[212] Shimobayashi M, Hall MN (2014). Making new contacts: the mTOR network in metabolism and signalling crosstalk. Nat Rev Mol Cell Biol.

[213] González A, Hall MN (2017). Nutrient sensing and TOR signaling in yeast and mammals. EMBO J.

[214] Hay N, Sonenberg N (2004). Upstream and downstream of mTOR. Genes Dev.

[215] Matsumoto CS, Almeida LO, Guimarães DM, Martins MD, Papagerakis P, Papagerakis S (2016). PI3K-PTEN dysregulation leads to mTOR-driven upregulation of the core clock gene BMAL1 in normal and malignant epithelial cells. Oncotarget.

[216] Bobrovnikova-Marjon E, Grigoriadou C, Pytel D, Zhang F, Ye J, Koumenis C (2010). PERK promotes cancer cell proliferation and tumor growth by limiting oxidative DNA damage. Oncogene.

[217] Pytel D, Gao Y, Mackiewicz K, Katlinskaya YV, Staschke KA, Paredes MCG (2016). PERK is a haploinsufficient tumor suppressor: gene dose determines tumor-suppressive versus tumor promoting properties of PERK in melanoma. PLoS Genet.

[218] Bhattacharya S, HuangFu WC, Dong G, Qian J, Baker DP, Karar J (2013). Anti-tumorigenic effects of Type 1 interferon are subdued by integrated stress responses. Oncogene.

[219] Lee Y, Fong SY, Shon J, Zhang SL, Brooks R, Lahens NF (2021). Time-of-day specificity of anticancer drugs may be mediated by circadian regulation of the cell cycle. Sci. Adv..

[220] Isakoff MS, Bielack SS, Meltzer P, Gorlick R (2015). Osteosarcoma: current treatment and a collaborative pathway to success. J Clin Oncol.

[221] Tang QL, Xie XB, Wang J, Chen Q, Han AJ, Zou CY (2012). Glycogen synthase kinase-3β, NF-κB signaling, and tumorigenesis of human osteosarcoma. J Natl Cancer Ins.

[222] Liu HC, Enikolopov G, Chen Y (2012). Cul4B regulates neural progenitor cell growth. BMC Neurosci.

[223] Soucy TA, Smith PG, Milhollen MA, Berger AJ, Gavin JM, Adhikari S (2009). An inhibitor of NEDD8-activating enzyme as a new approach to treat cancer. Nature.

[224] Nawrocki ST, Griffin P, Kelly KR, Carew JS (2012). MLN4924: a novel first-in-class inhibitor of NEDD8-activating enzyme for cancer therapy. Expert Opin Investig Drugs.

[225] Khalife J, Radomska HS, Santhanam R, Huang X, Neviani P, Saultz J (2015). Pharmacological targeting of miR-155 via the NEDD8-activating enzyme inhibitor MLN4924 (Pevonedistat) in FLT3-ITD acute myeloid leukemia. Leukemia.

[226] Chen P, Hu T, Liang Y, Jiang Y, Pan Y, Li C (2015). Synergistic inhibition of autophagy and neddylation pathways as a novel therapeutic approach for targeting liver cancer. Oncotarget.

[227] Kuo KL, Ho IL, Shi CS, Wu JT, Lin WC, Tsai YC (2015). MLN4924, a novel protein neddylation inhibitor, suppresses proliferation and migration of human urothelial carcinoma: In vitro and in vivo studies. Cancer Lett.

[228] Zhang S, Zhang J, Deng Z, Liu H, Mao W, Jiang F (2016). Circadian clock components RORα and Bmal1 mediate the anti-proliferative effect of MLN4924 in osteosarcoma cells. Oncotarget.

[229] Yu X, Rollins D, Ruhn KA, Stubblefield JJ, Green CB, Kashiwada M (2013). TH17 cell differentiation is regulated by the circadian clock. Science.

[230] Hand LE, Gray KJ, Dickson SH, Simpkins DA, Ray DW, Konkel JE (2020). Regulatory T cells confer a circadian signature on inflammatory arthritis. Nat Commun.

[231] Hu X, Liu X, Moisan J, Wang Y, Lesch CA, Spooner C (2016). Synthetic RORγ agonists regulate multiple pathways to enhance antitumor immunity. Oncoimmunology.

[232] Mullins D, Proulx D, Saoudi A, Ng CE (2005). Chronomodulation of topotecan or X-radiation treatment increases treatment efficacy without enhancing acute toxicity. Int J Radiat Oncol Biol Phys.

[233] Ozturk N, Ozturk D, Pala-Kara Z, Kaptan E, Sancar-Bas S, Ozsoy N (2018). The immune system as a chronotoxicity target of the anticancer mTOR inhibitor everolimus. Chronobiol Int.

