# Supplementary material for: Major roles of the circadian clock in cancer
Source: Cancer Biol Med. 2023 Jan 12;20(1):1–24. doi: 10.20892/j.issn.2095-3941.2022.0474 (PMC9843445; doi:10.20892/j.issn.2095-3941.2022.0474)
Supplement: Supplementary file 1 [file cbm-20-001-s001.pdf]

Supplementary materials

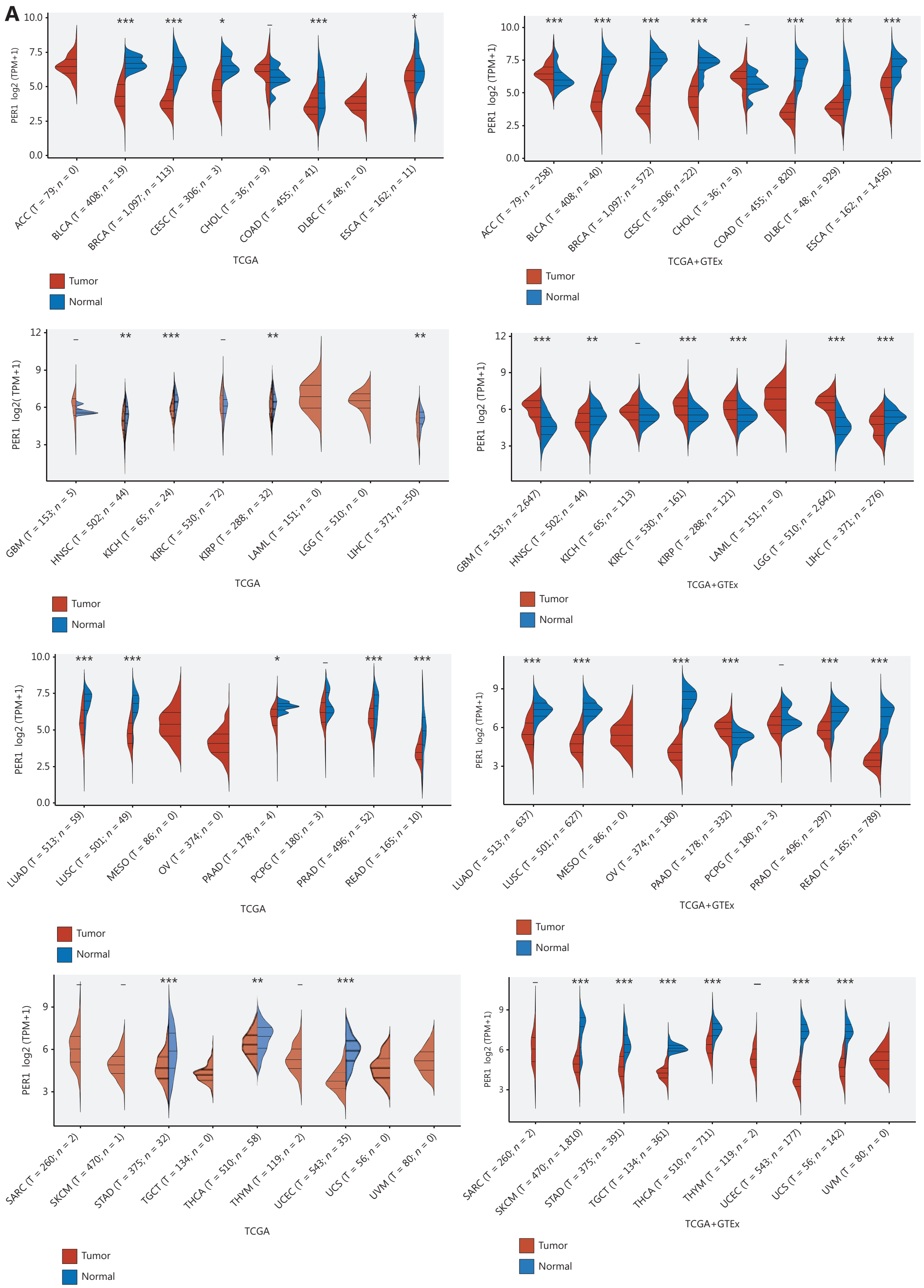

Figure S1 Continued

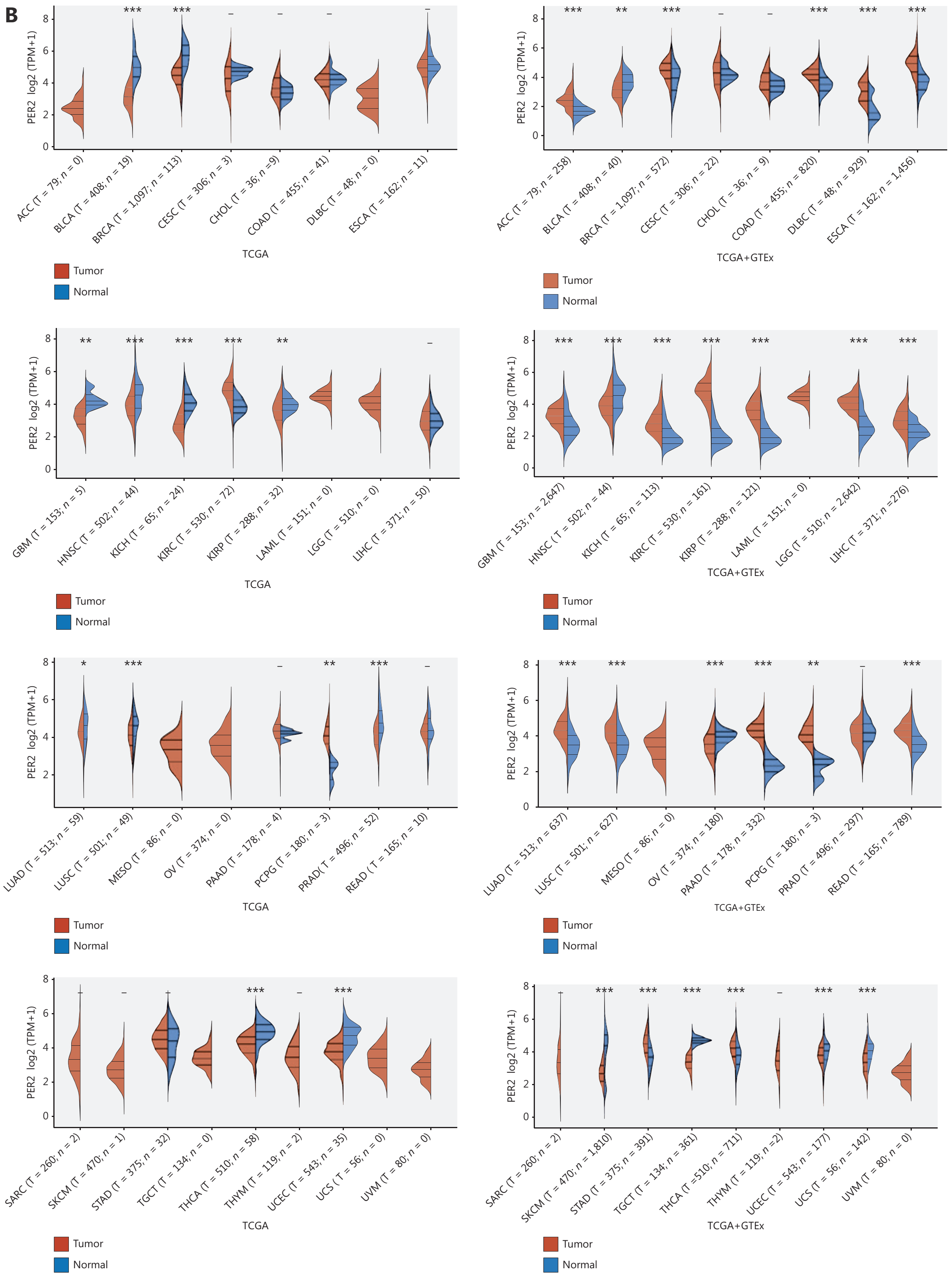

Figure S1 Continued

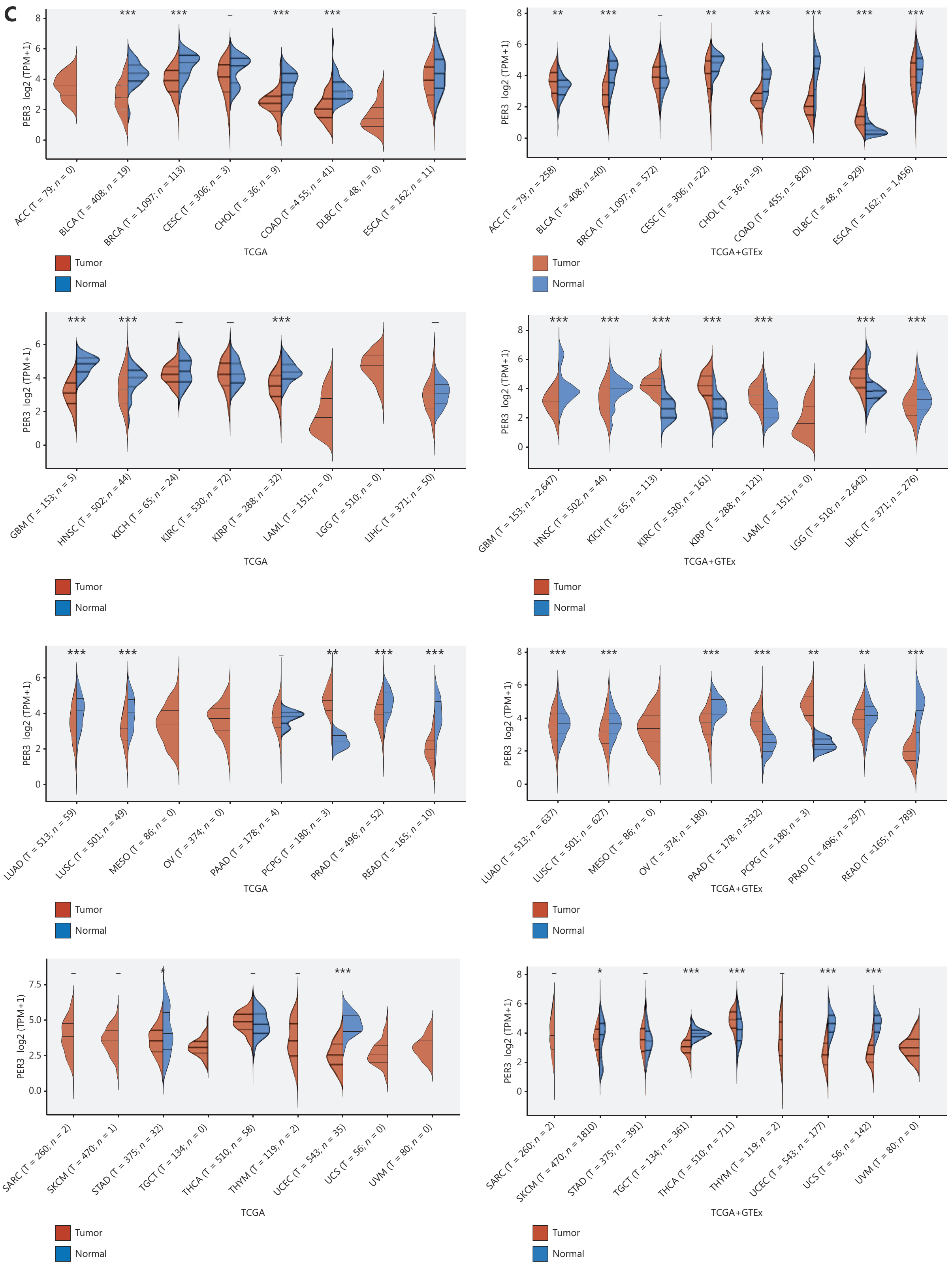

Figure S1 Expression analysis of PER in tumor tissues. (A) PER1, (B) PER2, (C) PER3.

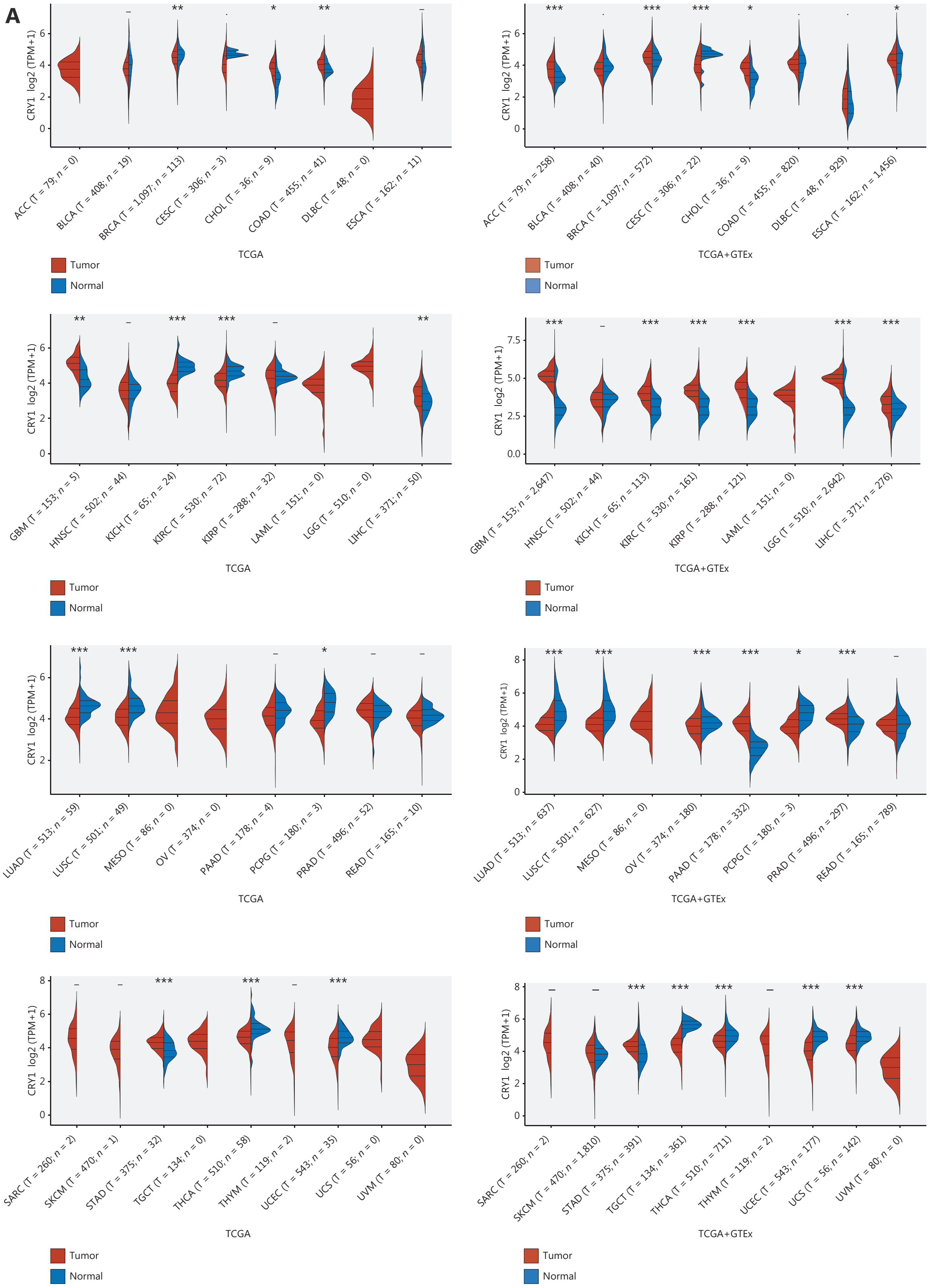

Figure S2 Continued

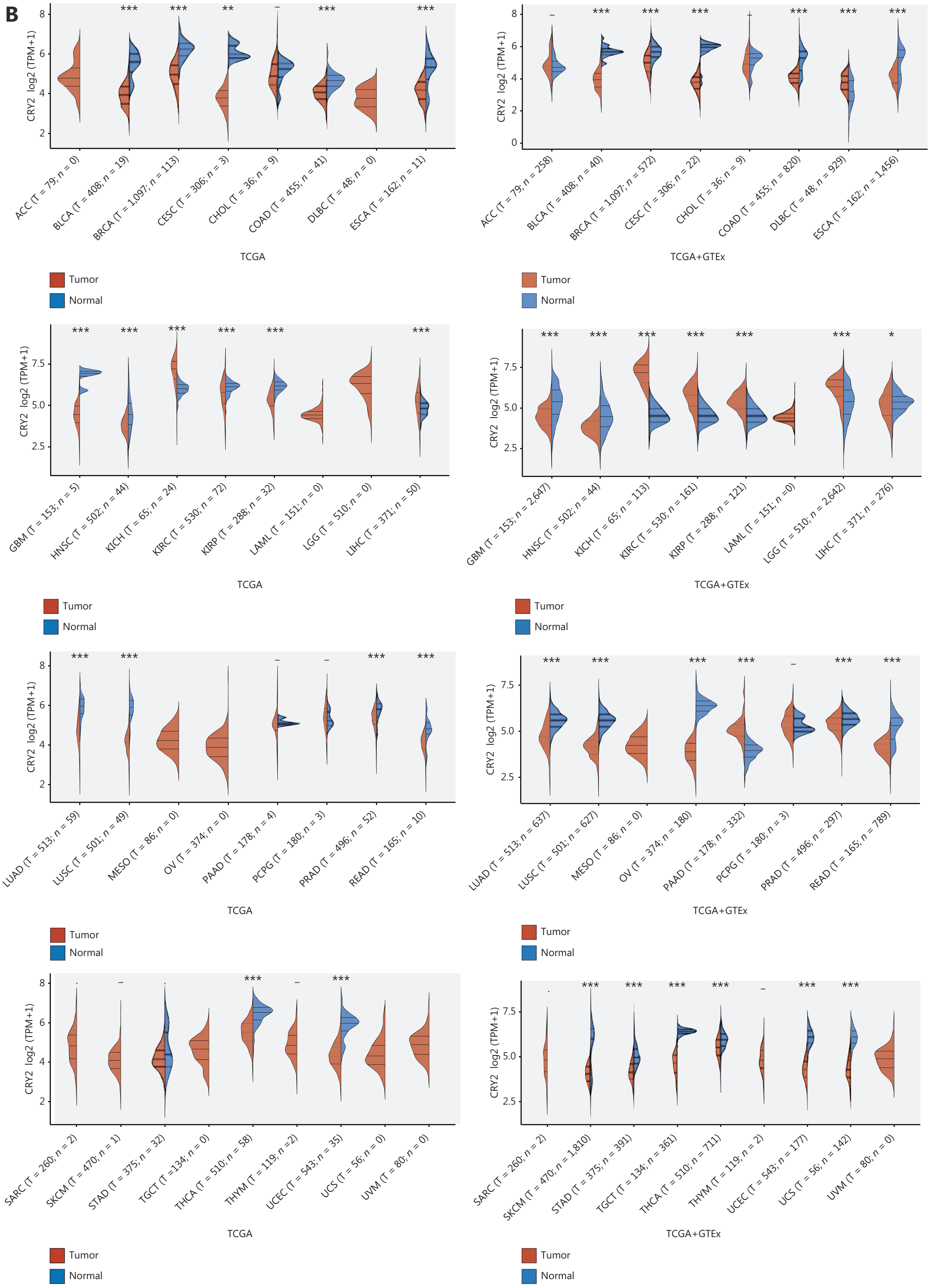

Figure S2 Expression analysis of CRY in tumor tissues. (A) CRY1, (B) CRY2.

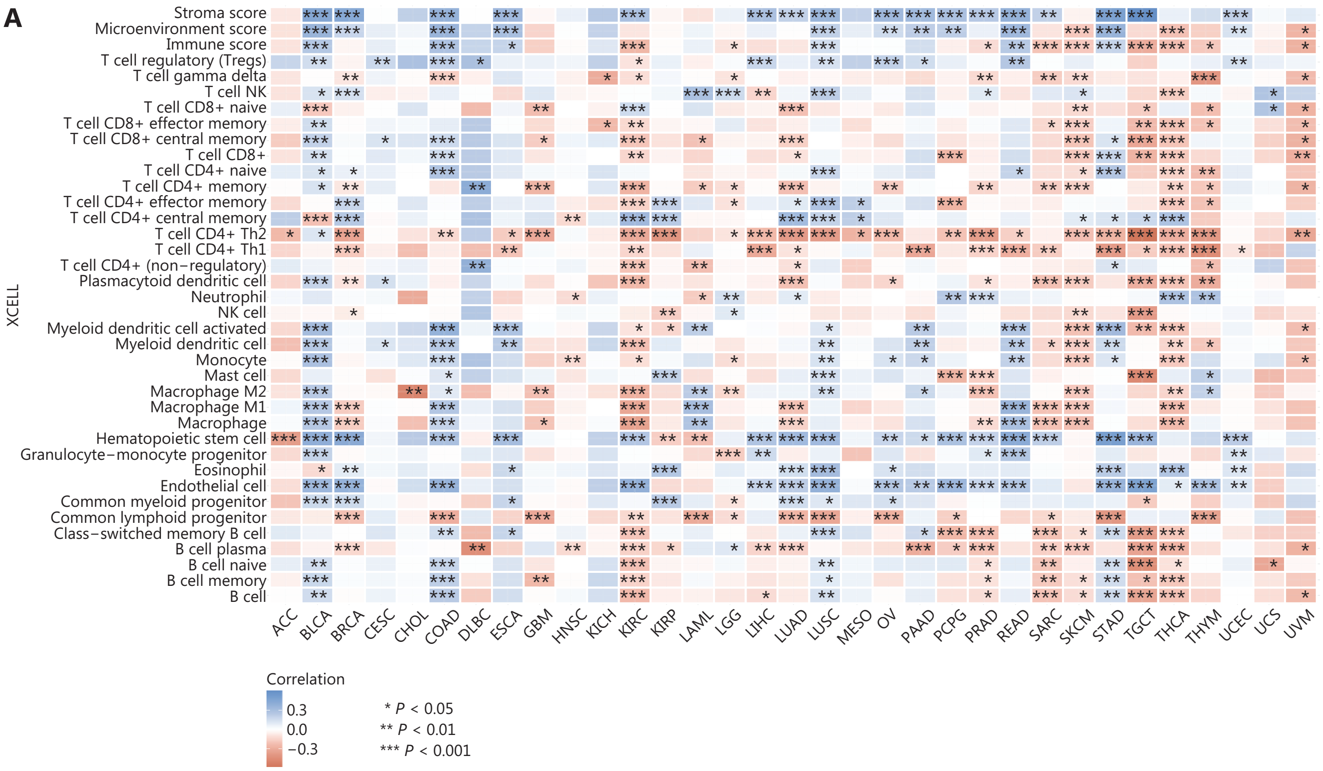

Figure S3 Continued

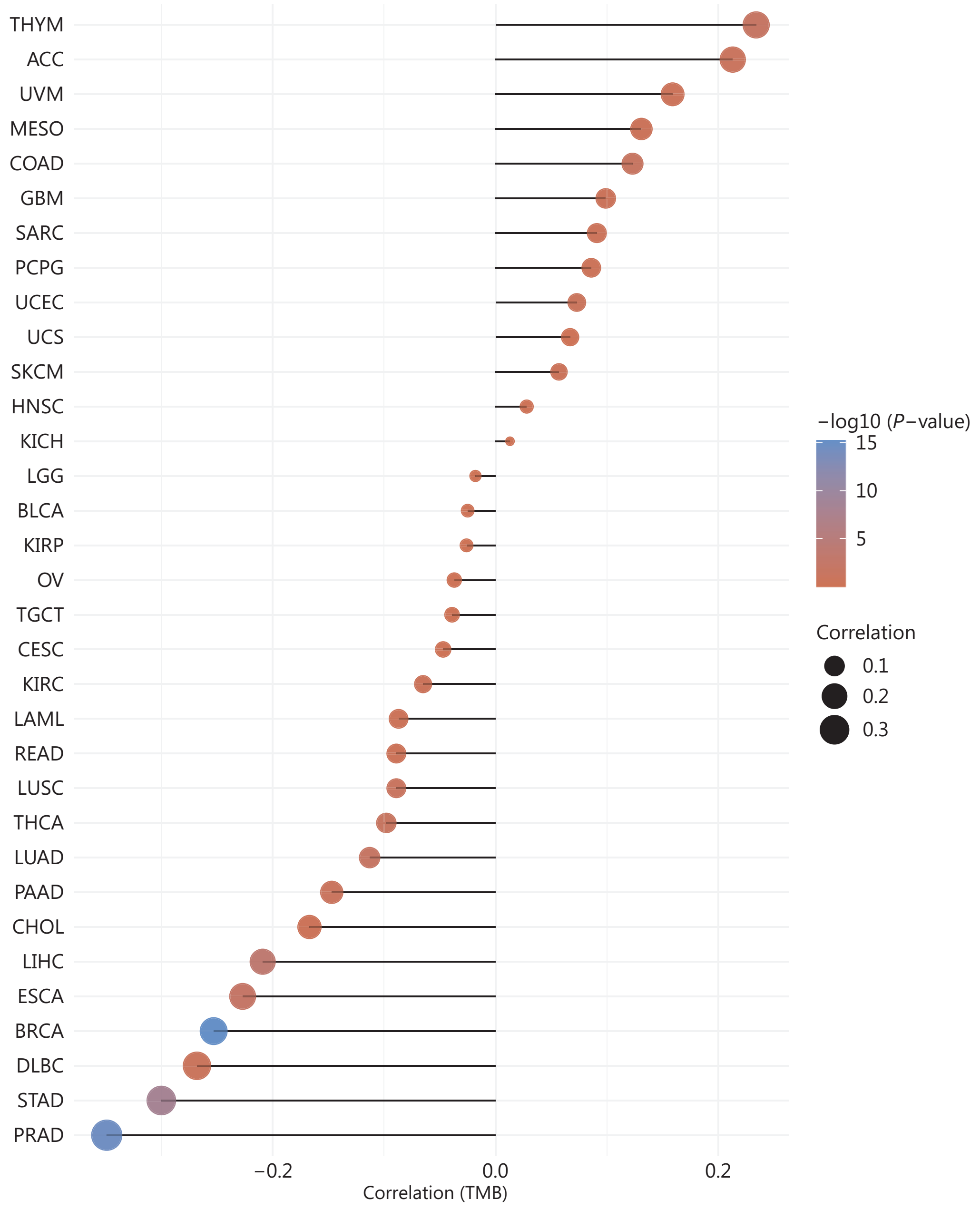

Figure S3 Continued

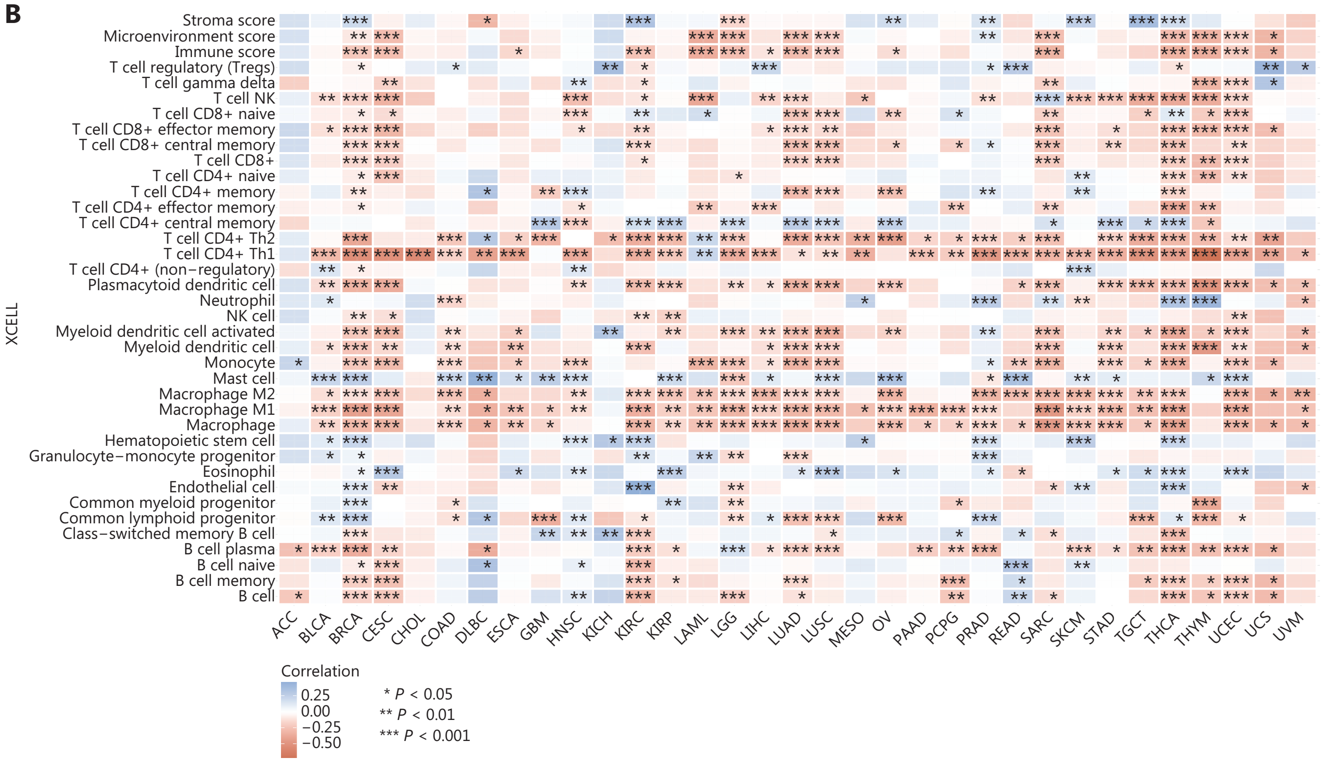

Figure S3 Continued

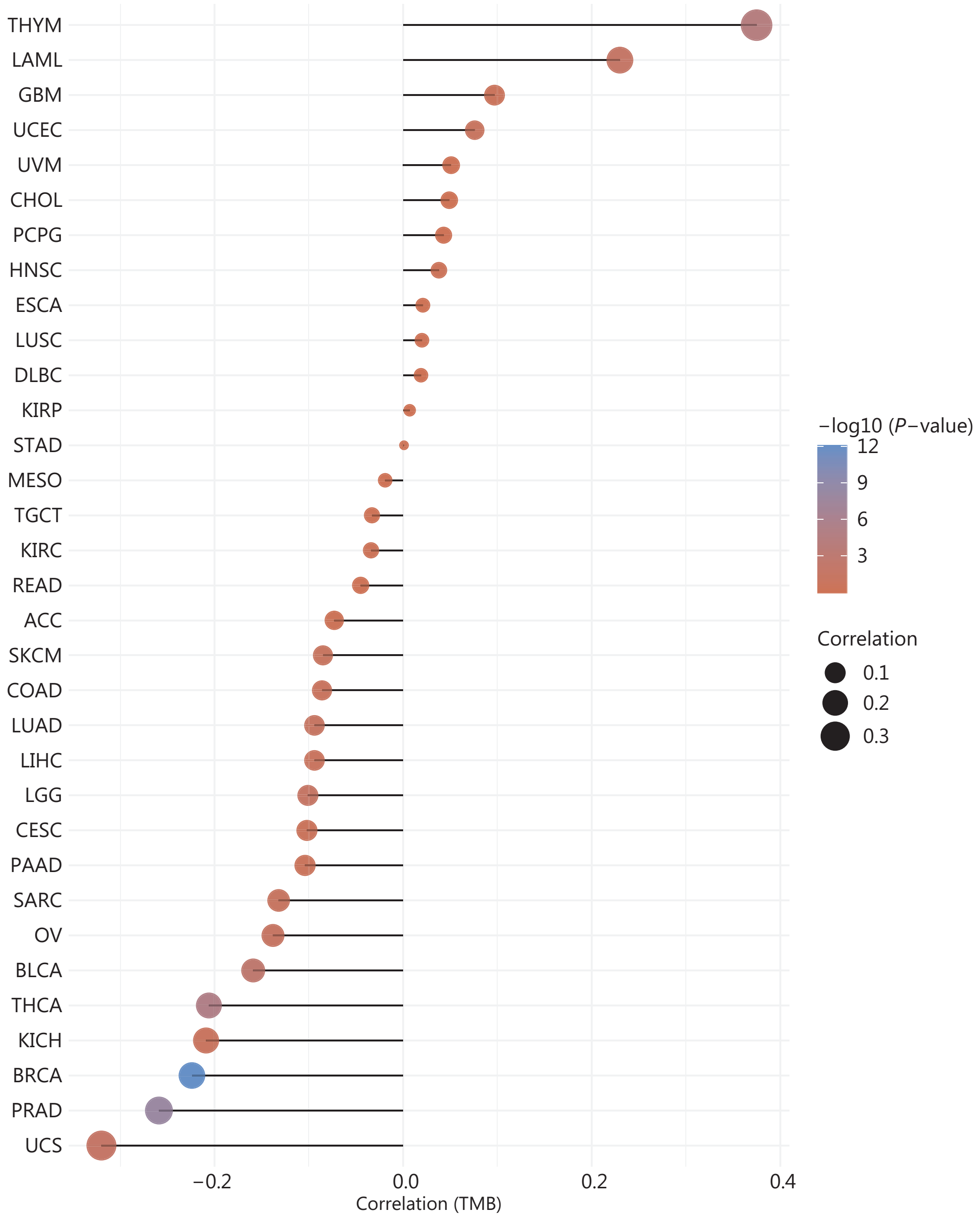

Figure S3 Continued

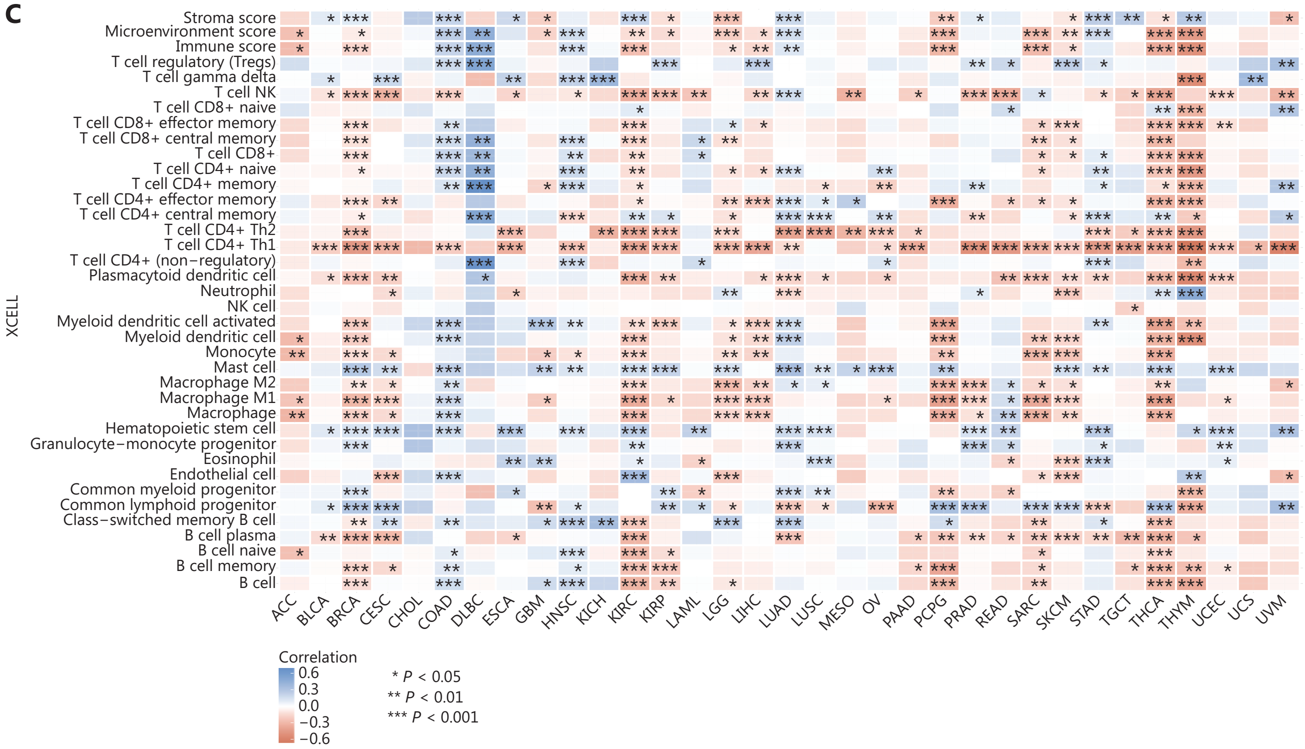

Figure S3 Continued

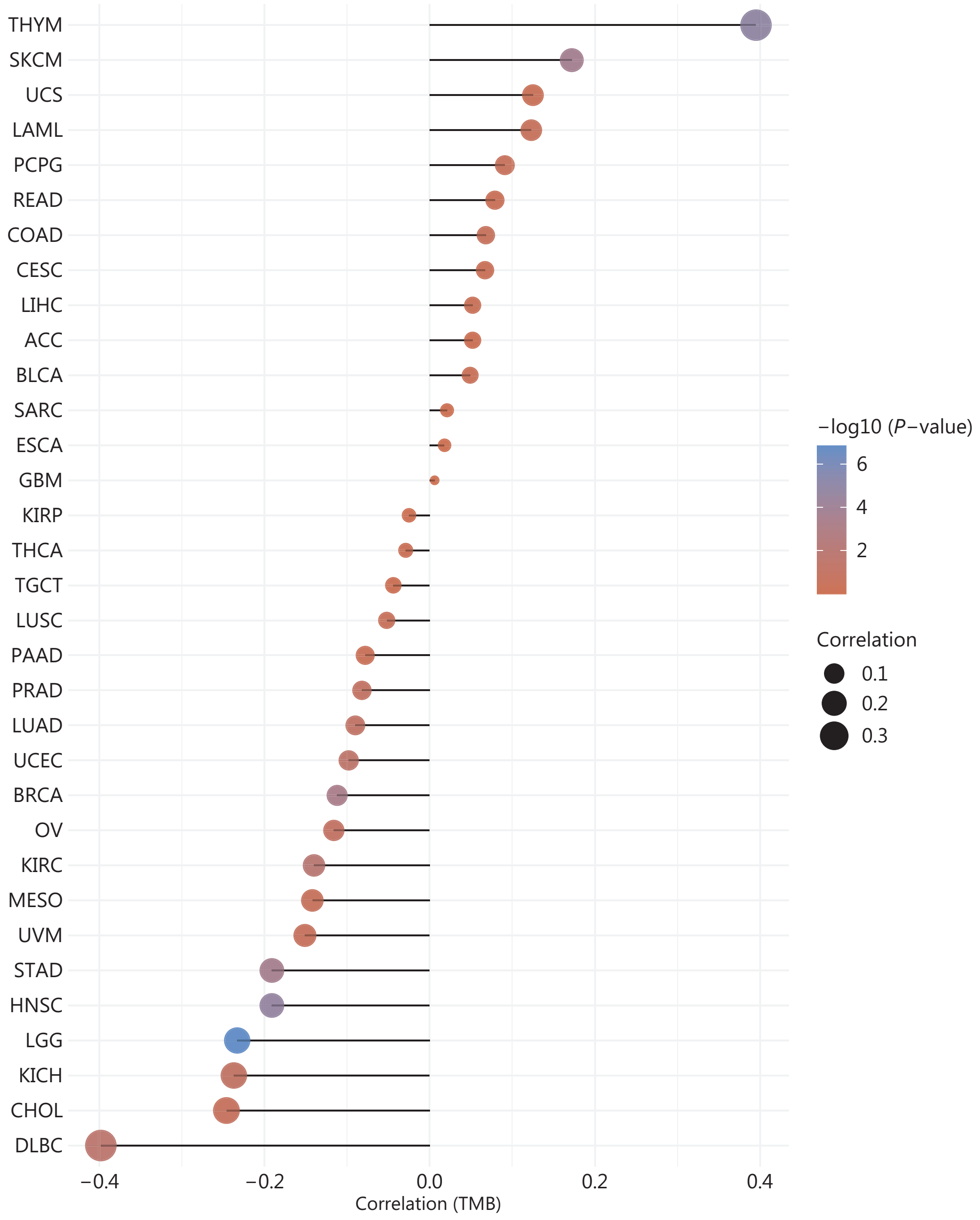

Figure S3 Immune-associated analysis and tumor mutational burden of PER. (A) PER1, (B) PER2, (C) PER3.

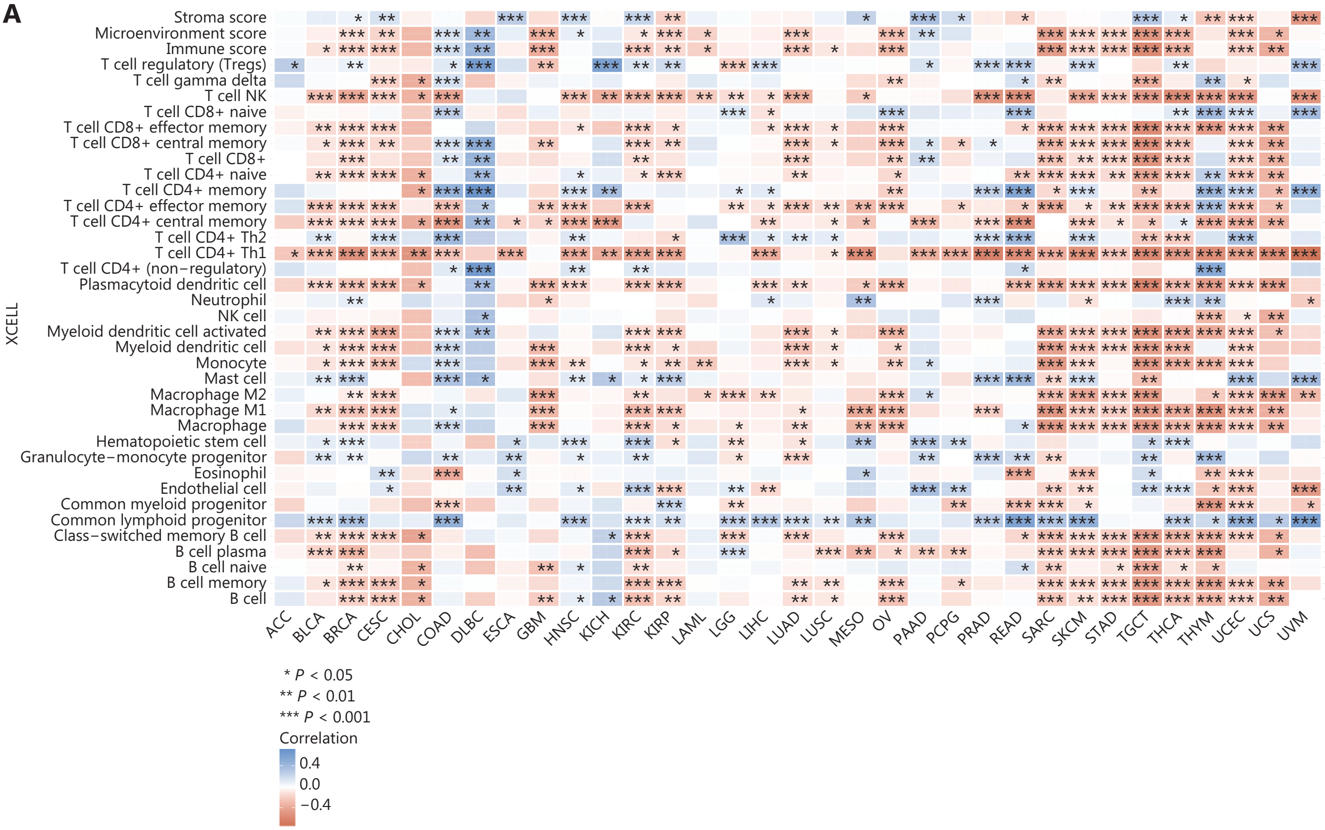

Figure S4 Continued

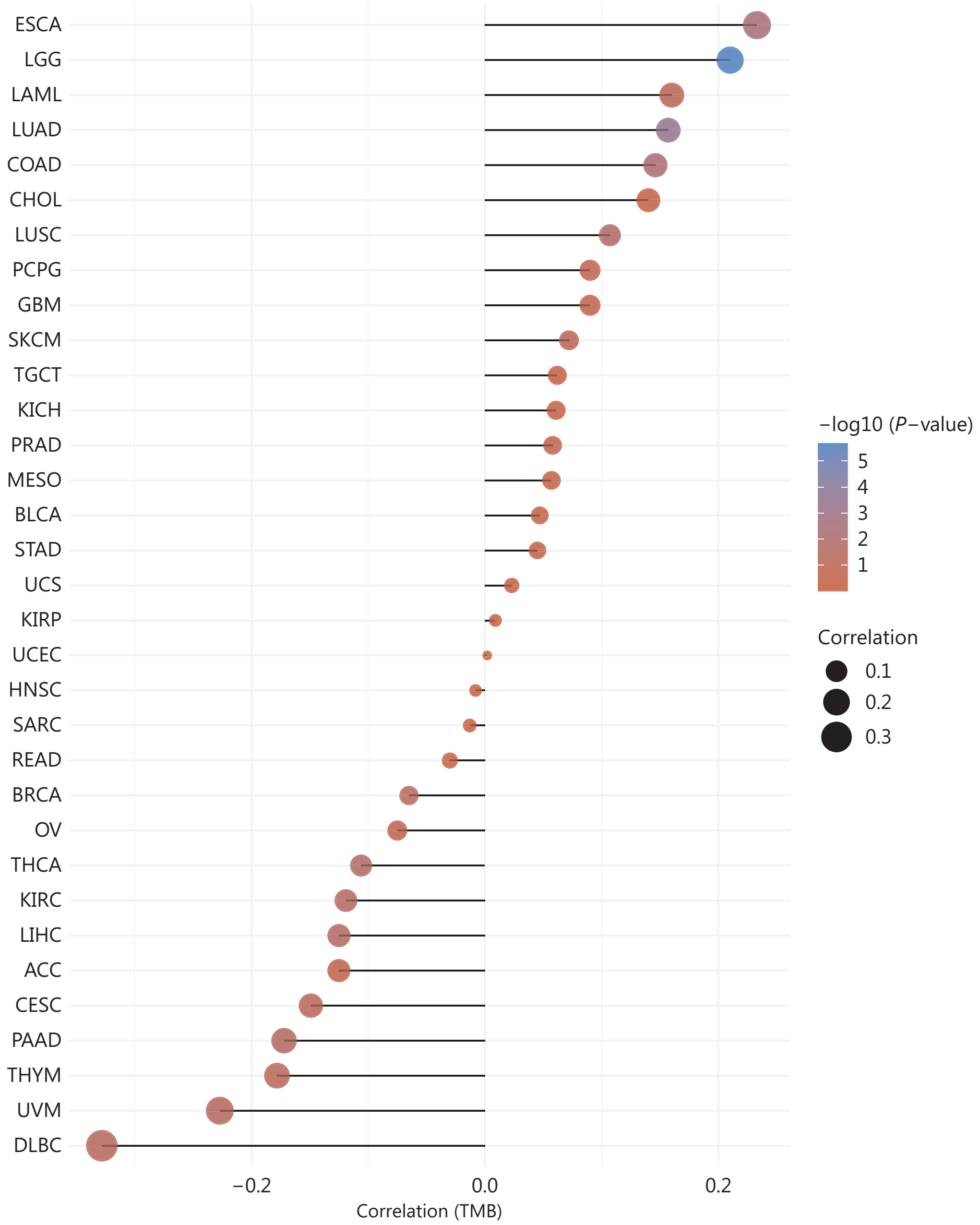

Figure S4 Continued

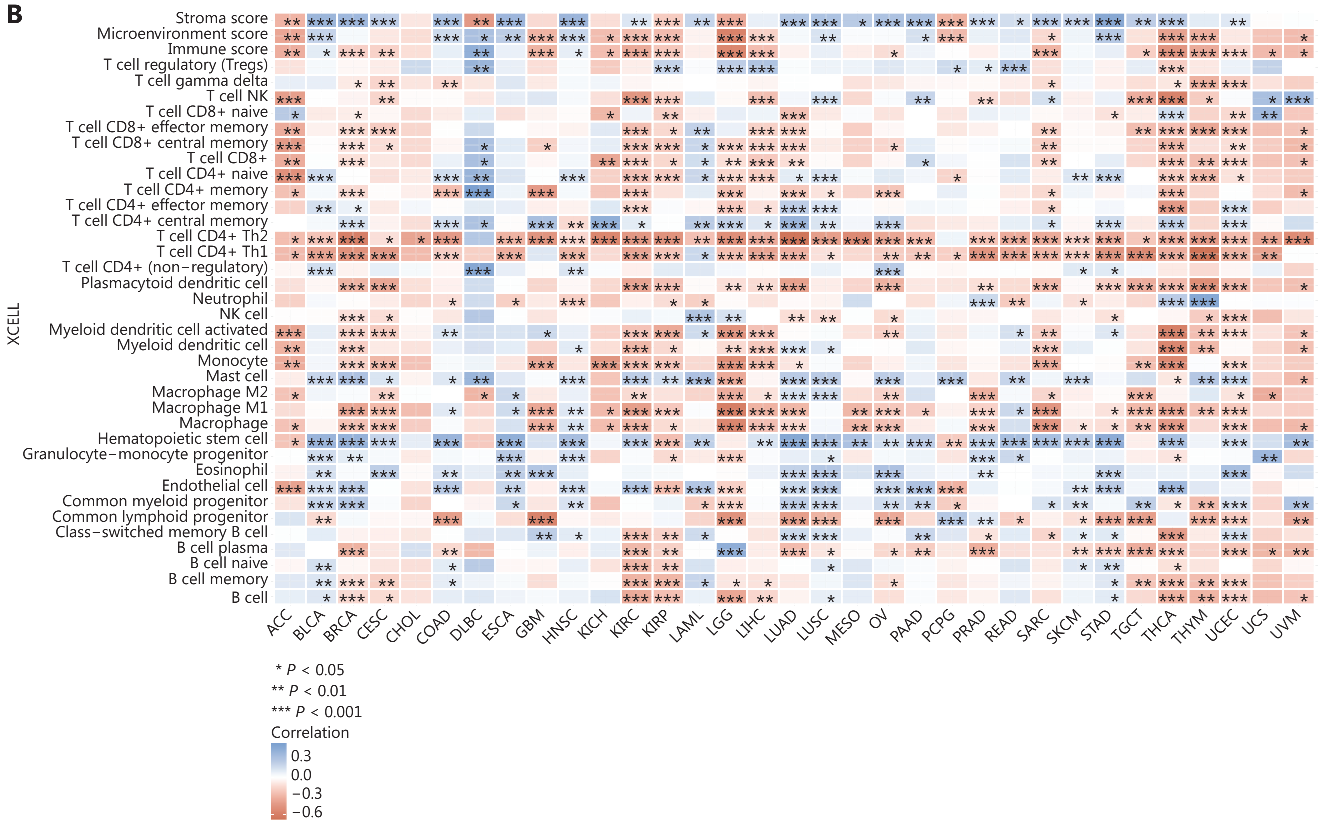

Figure S4 Continued

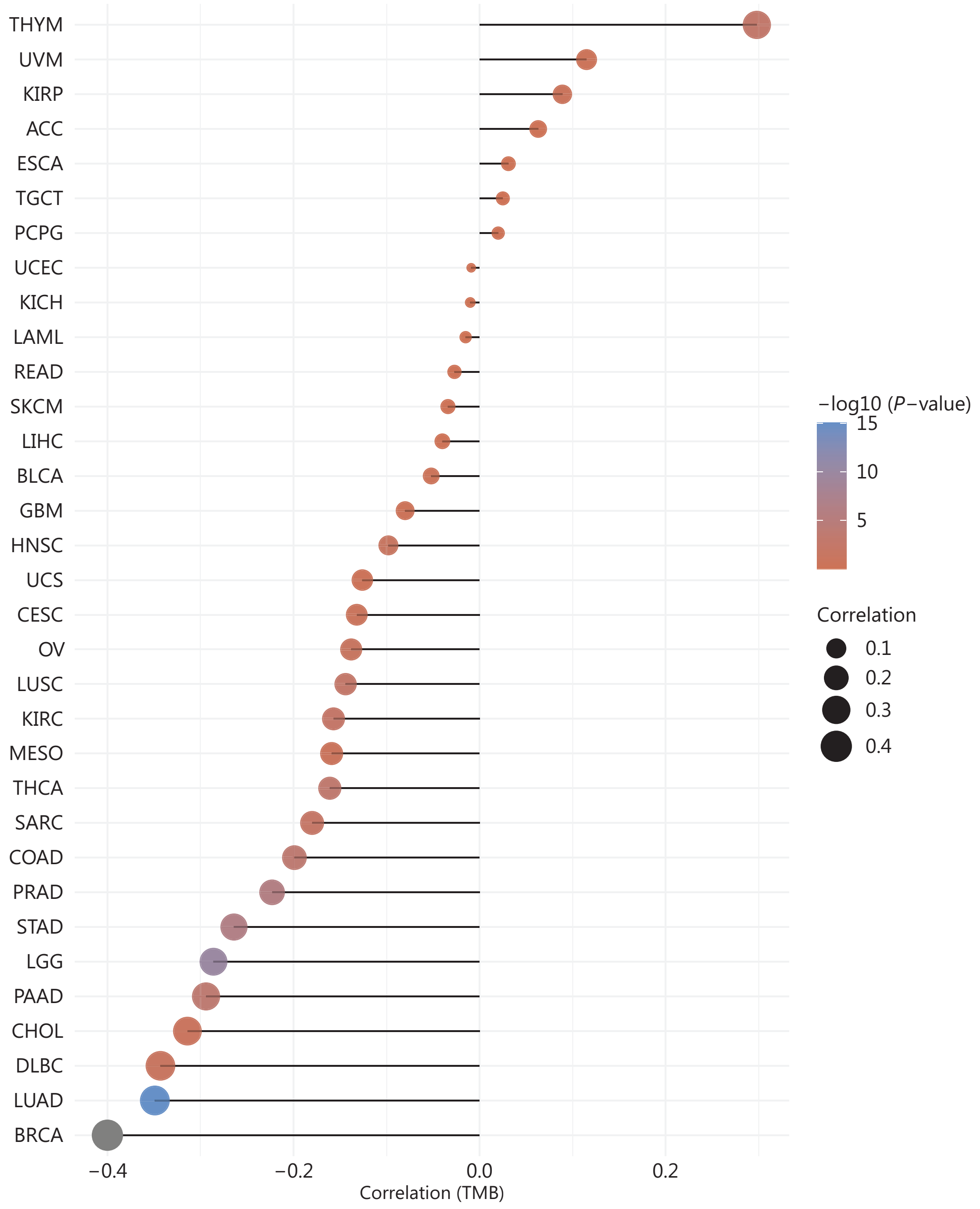

Figure S4 Immune-associated analysis and tumor mutational burden of CRY. (A) CRY1, (B) CRY2.

A

Cancer	*P*-value	Hazard ratio (95% CI)
ACC	0.7880	1.10774 (0.52555, 2.33487)
BLCA	0.4174	0.88544 (0.65986, 1.18813)
BRCA	0.4722	0.89011 (0.64804, 1.22261)
CESC	0.0954	0.67226 (0.42153, 1.07212)
CHOL	0.1079	2.27589 (0.83512, 6.20229)
COAD	0.2207	1.27622 (0.8638, 1.88557)
DLBC	0.1012	3.95017(0.7642,20.41865)
ESCA	0.0492	0.60653(0.36856,0.99817)
GBM	0.8886	1.02596 (0.71679, 1.46849)
HNSC	0.2711	0.86086 (0.65925, 1.12411)
KICH	0.0993	0.26661 (0.05536, 1.28398)
KIRC	0.1047	0.77763 (0.57391, 1.05367)
KIRP	0.0023	0.34524 (0.17439, 0.68346)
LAML	0.6860	0.91637 (0.60004, 1.39946)
LGG	0.2387	1.2358 (0.86898, 1.75745)
LIHC	0.0135	0.64499 (0.45551, 0.91329)
LUAD	0.5117	1.10207 (0.8244, 1.47327)
LUSC	0.2903	1.15846 (0.88204, 1.52151)
MESO	0.3378	0.79433 (0.49606, 1.27193)
OV	0.0519	1.29512 (0.99786, 1.68093)
PAAD	0.7185	0.9273 (0.61521, 1.39771)
PCPG	0.6946	1.35112 (0.30075, 6.06998)
PRAD	0.4335	0.57632 (0.14512, 2.28876)
READ	0.9202	0.96048 (0.4363, 2.11444)
SARC	0.6805	1.08659 (0.73185, 1.61328)
SKCM	0.5367	1.08811 (0.83239, 1.4224)
STAD	0.0095	1.55828 (1.11445, 2.17888)
TGCT	0.3659	2.8416 (0.29544, 27.33135)
THCA	0.3659	1.5956 (0.57952, 4.39318)
THYM	0.1121	3.16147 (0.76423, 13.0784)
UCEC	0.1132	1.39921 (0.9234, 2.12021)
UCS	0.6941	0.86858 (0.43035, 1.75305)
UVM	0.2636	0.62404 (0.27303, 1.42632)

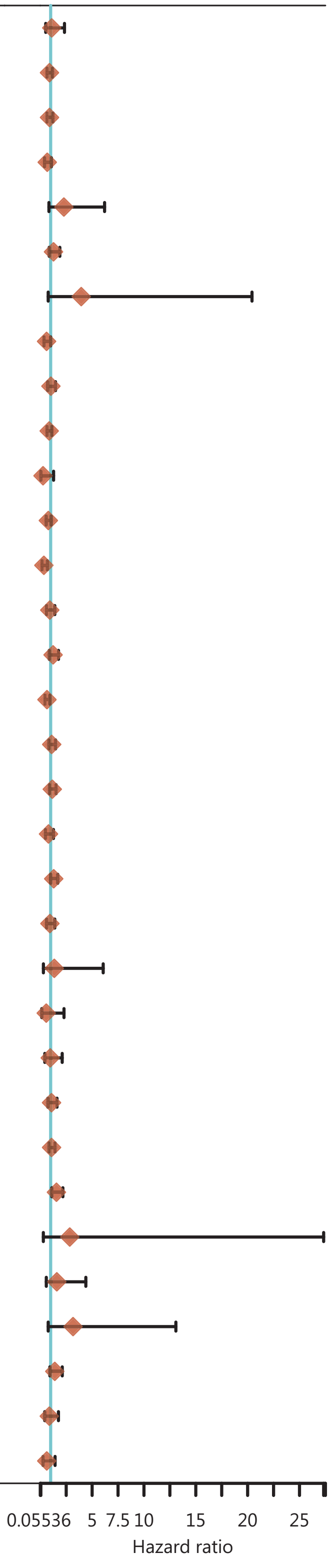

Figure S5 Continued

B

Cancer	*P*-value	Hazard ratio (95% CI)
ACC	0.8527	0.85903 (0.17281, 4.27016)
BRCA	0.4747	0.61454 (0.16171, 2.33542)
HNSC	0.0353	0.5115 (0.27398, 0.95494)
LGG	0.0690	0.54492 (0.2832, 1.04848)
LUAD	0.9550	1.04458 (0.22966, 4.75114)
LUSC	0.5101	0.64094 (0.17058, 2.40824)
MESO	0.4817	1.38949 (0.55581, 3.47362)
PAAD	0.9637	1.02931 (0.29665, 3.57149)
SARC	0.5159	1.28647 (0.60168, 2.75066)
SKCM	0.6723	1.14375 (0.61388, 2.13098)
STAD	0.0893	0.41597 (0.15123, 1.14418)
THCA	0.4347	1.738 (0.43433, 6.95475)
THYM	0.1857	0.28526 (0.0445, 1.82853)
UCEC	0.5085	2.26458 (0.20071, 25.55113)
UCS	0.8491	0.89534 (0.28666, 2.79646)

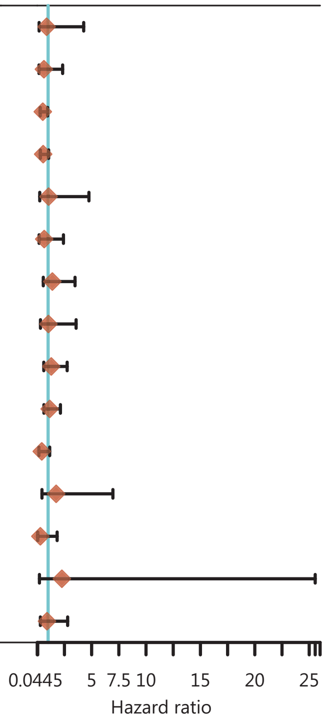

C

Cancer	*P*-value	Hazard ratio (95% CI)
ACC	0.9067	0.90862 (0.18297, 4.51216)
BRCA	0.1977	0.35338 (0.07258, 1.72056)
HNSC	0.0685	0.57127 (0.3128, 1.04332)
LGG	0.0002	0.25216 (0.12112, 0.52498)
LUAD	0.7852	0.81188 (0.18142, 3.63339)
LUSC	0.0254	6.5897 (1.261, 34.43636)
MESO	0.2335	0.5636 (0.21943, 1.44756)
PAAD	0.6010	1.40442 (0.39323, 5.01591)
SARC	0.5792	0.80608 (0.37628, 1.72683)
SKCM	0.3379	1.36764 (0.72091, 2.59454)
STAD	0.2957	0.58983 (0.21926, 1.5867)
THCA	0.3176	2.02834 (0.5067, 8.11957)
THYM	0.8366	0.82383 (0.1306, 5.1967)
UCEC	0.4360	2.6044 (0.23428, 28.95256)
UCS	0.5686	1.41547 (0.42871,4.67343)

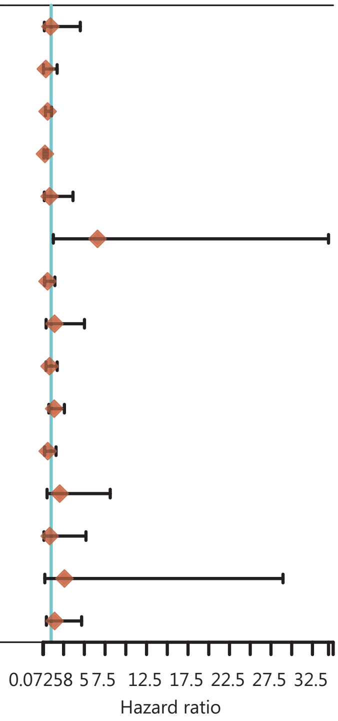

Figure S5 PER and prognosis of cancer. (A) PER1, (B) PER2, (C) PER3.

A

Cancer	*P*-value	Hazard ratio (95% CI)
ACC	0.4895	1.82069 (0.33278, 9.96139)
BRCA	0.9659	1.02961 (0.27061,3 .91738)
HNSC	0.7712	0.91466 (0.50134, 1.66873)
LGG	0.2698	1.45146 (0.74888, 2.81316)
LUAD	0.2083	0.34114 (0.06387, 1.82201)
LUSC	0.4510	1.71445 (0.422, 6.96526)
MESO	0.0585	2.67248 (0.96525, 7.39931)
PAAD	0.6655	1.31674 (0.37824, 4.58393)
SARC	0.1493	1.75009 (0.81787, 3.74487)
SKCM	0.3814	1.32228 (0.70739, 2.47164)
STAD	0.6904	1.21378 (0.46785, 3.14901)
THCA	0.2199	2.38416 (0.59502, 9.55296)
THYM	0.0332	0.08168 (0.00814, 0.81925)
UCEC	0.5449	2.10652 (0.18883, 23.49959)
UCS	0.3989	1.64045 (0.51948, 5.18032)

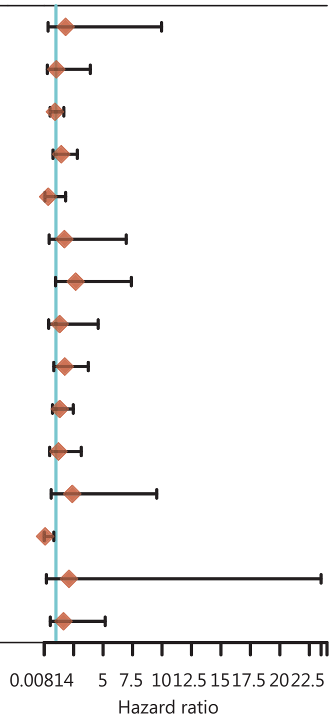

B

Cancer	*P*-value	Hazard ratio (95% CI)
ACC	0.3315	0.42931 (0.0779, 2.36592)
BRCA	0.9205	1.07343 (0.2672, 4.31229)
HNSC	0.8969	0.96054 (0.52247, 1.76594)
LGG	0.0020	0.31557 (0.15177, 0.65613)
LUAD	0.0829	0.14796 (0.01707, 1.28234)
LUSC	0.5788	0.63702 (0.12961, 3.13097)
MESO	0.8801	0.93232 (0.37514, 2.31705)
PAAD	0.1024	0.32155 (0.08241, 1.25464)
SARC	0.5383	0.79168 (0.37623, 1.66592)
SKCM	0.5907	1.18928 (0.63229, 2.23693)
STAD	0.5766	1.3152 (0.50265, 3.44127)
THCA	0.4101	1.79203 (0.44717, 7.18153)
THYM	0.9807	1.02261 (0.16742, 6.24616)
UCEC	0.7737	0.7014 (0.06252, 7.86928)
UCS	0.9585	1.03055 (0.33137, 3.20495)

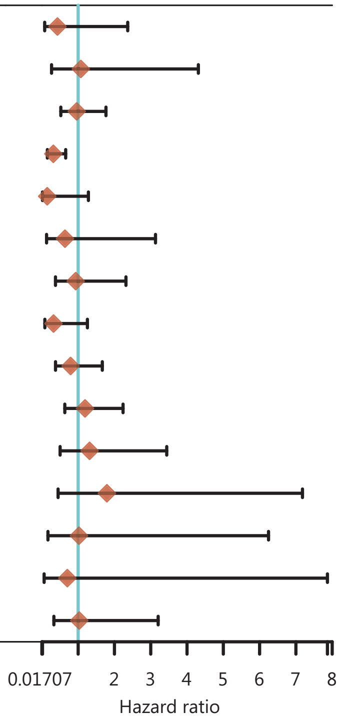

Figure S6 CRY and prognosis of cancer. (A) CRY1, (B) CRY2.
